# Bacteriome from *Pinus arizonica* and *P. durangensis*: Diversity, Comparison of Assemblages, and Overlapping Degree with the Gut Bacterial Community of a Bark Beetle That Kills Pines

**DOI:** 10.3389/fmicb.2018.00077

**Published:** 2018-01-30

**Authors:** Roman Gonzalez-Escobedo, Carlos I. Briones-Roblero, Rosa M. Pineda-Mendoza, Flor N. Rivera-Orduña, Gerardo Zúñiga

**Affiliations:** ^1^Laboratorio de Variación Biológica y Evolución, Departamento de Zoología, Escuela Nacional de Ciencias Biológicas, Instituto Politécnico Nacional, Mexico City, Mexico; ^2^Laboratorio de Ecología Microbiana, Departamento de Microbiología, Escuela Nacional de Ciencias Biológicas, Instituto Politécnico Nacional, Mexico City, Mexico

**Keywords:** endophytic bacteria, Arizona pine, Durango pine, 16S rRNA gene pyrosequencing, bark beetle endomicrobiome

## Abstract

Symbioses between plants and microorganims have been fundamental in the evolution of both groups. The endophytic bacteria associated with conifers have been poorly studied in terms of diversity, ecology, and function. Coniferous trees of the genera *Larix*, *Pseudotsugae*, *Picea* and mainly *Pinus*, are hosts of many insects, including bark beetles and especially the *Dendroctonus* species. These insects colonize and kill these trees during their life cycle. Several bacteria detected in the gut and cuticle of these insects have been identified as endophytes in conifers. In this study, we characterized and compared the endophytic bacterial diversity in roots, phloem and bark of non-attacked saplings of *Pinus arizonica* and *P. durangensis* using 16S rRNA gene pyrosequencing. In addition, we evaluated the degree of taxonomic relatedness, and the association of metabolic function profiles of communities of endophytic bacteria and previously reported gut bacterial communities of *D. rhizophagus*; a specialized bark beetle that colonizes and kills saplings of these pine species. Our results showed that both pine species share a similar endophytic community. A total of seven bacterial phyla, 14 classes, 26 orders, 43 families, and 51 genera were identified. Enterobacteriaceae was the most abundant family across all samples, followed by Acetobacteraceae and Acidobacteriaceae, which agree with previous studies performed in other pines and conifers. Endophytic communities and that of the insect gut were significantly different, however, the taxonomic relatedness of certain bacterial genera of pines and insect assemblages suggested that some bacteria from pine tissues might be the same as those in the insect gut. Lastly, the metabolic profile using PICRUSt showed there to be a positive association between communities of both pines and insect gut. This study represents the baseline into the knowledge of the endophytic bacterial communities of two of the major hosts affected by *D. rhizophagus*.

## Introduction

Symbiosis has been recognized as a “key driver” force in the species evolutionary process. Plant-microorganism interactions have resulted in beneficial, neutral or detrimental effects for both groups. In particular, bacterial communities carry out different biological activities in organs and tissues of plants: rhizosphere, phyllosphere, and endosphere (Turner et al., 2013). Endophytic microorganisms of the endosphere, i.e., those that reside within the inner tissues of plants without causing any symptoms of disease (Hallmann et al., 1997), have enhanced plant tolerance to different biotic and abiotic factors and mediated the interaction with parasites (Reinhold-Hurek and Hurek, 2011; Hardoim et al., 2015; Hashem et al., 2016).

Research on microbial endophytes in plants has been mainly focused on crops of economic importance (e.g., corn, sorghum, soybean, wheat) (Zinniel et al., 2002), but little attention has been paid to wild plants, especially conifers (Carrell and Frank, 2014). A number of studies in conifers have partially characterized the endophytic diversity in different tissues (roots, stems, buds, needles, seeds, and pollen), but almost none have explored the ecological and functional role of these bacteria (O’Neill et al., 1992; Shishido et al., 1995; Pirttilä et al., 2000; Strzelczyk and Li, 2000; Cankar et al., 2005; Izumi et al., 2008; Bal et al., 2012). In addition, most of these studies have been carried out using culture-dependent methods and traditional molecular techniques (e.g., molecular cloning, fingerprinting). Given that these approaches are limited in their statistical coverage, they can lead to biases in diversity characterization by the low number of sequences recovered. In contrast, the application of next generation sequencing (NGS) technologies overcomes these limitations and allows a better taxonomic and functional characterization of endophytic communities in conifers (Carrell and Frank, 2014, 2015; Carrell et al., 2016; Kaul et al., 2016; Rúa et al., 2016).

The *Larix*, *Pseudotsugae*, *Picea*, and *Pinus* genera are hosts of a group of weevils, *Dendroctonus* bark beetles, because they carry out their life cycle inside trees. These insects play an important role in coniferous forests as agents of natural renovation and regeneration, but they are also one of the most destructive insects of these communities because they kill from 100 to 1000s of healthy trees during periodic outbreaks (Wood, 1982; Reeve et al., 2012). The life cycle of these bark beetles is apparently simple. Females locate and arrive on trees following host kairomones (Gray et al., 2015). Later, they bore the bark toward the phloem, and once inside produce pheromones to attract males, sometimes leading to mass attacks on trees. Pairs copulate and excavate galleries where females oviposite. Then, larvae hatch, feed on the stem phloem, develop into new adults and lastly, emerge to colonize other pine trees (Raffa et al., 2008).

Several studies have documented the diversity and functional role of bacterial communities associated with certain *Dendroctonus* species and their galleries using different methodologies (Cardoza et al., 2006; Scott et al., 2008; Adams et al., 2009, 2013; Morales-Jiménez et al., 2009, 2012, 2013; Boone et al., 2013; Durand et al., 2015; Mason et al., 2015; Dohet et al., 2016; Xu L.T. et al., 2016; Xu L. et al., 2016; Briones-Roblero et al., 2017a,b). In addition, Hernández-García et al. (2017) determined the presence of a strict core bacteriome in the gut of these insects, constituted by *Enterobacter, Pantoea, Pseudomonas, Rahnella, Raoultella*, and *Serratia*, and a relaxed core composed of *Acinetobacter*, *Propionibacterium*, *Providencia*, *Stenotrophomonas*, *Erwinia*, *Kluyvera*, *Paenibacillus*, and *Ralstonia*. Several of these bacterial genera present in the gut and cuticle of *Dendroctonus* bark beetles have been reported as endophytes in pines and other coniferous genera (Pirttilä et al., 2000; Strzelczyk and Li, 2000; Cankar et al., 2005; Izumi et al., 2008; Bal et al., 2012; Carrell and Frank, 2015; Rúa et al., 2016), suggesting that these bacteria might be environmentally acquired, mainly during phloem feeding (Hernández-García et al., 2017).

In this study, we characterized and compared the endophytic bacterial diversity in roots, phloem and bark of non-attacked saplings of *Pinus arizonica* Engelm (Arizona pine) and *P. durangensis* Martinez (Durango pine), using 16S rRNA gene pyrosequencing. In addition, we explored the degree of taxonomic relatedness and the association of functional metabolic profile of endophytic bacterial communities and previously reported gut bacterial communities of the bark beetle, *D. rhizophagus* (Briones-Roblero et al., 2017a). These pine species were selected based on the fact that they are two preferential hosts of this insect which colonizes and kills saplings of 11 pine species, mainly Arizona and Durango pines, in Mexico (Salinas-Moreno et al., 2004; Mendoza et al., 2011). This is also because the gut bacterial community of this insect has been widely characterized and a core bacteriome has been determined from all life stages (Briones-Roblero et al., 2017a).

## Materials and Methods

### Sample Collection and Surface Sterilization

Healthy saplings of Arizona and Durango pines (<3 m tall, 10 cm diameter), non-attacked by *Dendroctonus rhizophagus*, were collected in San Juanito, Bocoyna Municipality, Chihuahua state (27° 45′ 11′′ N 107° 38′ 06′′ W, 2288 masl), and El Salto locality, Pueblo Nuevo Municipality, Durango state, Mexico (23° 41′ 31′′ N 105° 43′ 19′′ W, 2702 masl), respectively. Six trees of each pine species were removed from the soil using a peak and shovel, to integrate two biological replicates of three trees each one. Roots and stems were covered with plastic film, and stored at 4°C for transportation to the laboratory where they were immediately processed.

Plant parts were surface rinsed with sterile water for eliminating rhizospheric soil and contaminants. Several small fragments of approximately 2 cm × 3 cm of roots, phloem, and bark were obtained with sterile knife and fine forceps, placed in sterile resealable bags and stored at -20°C until processing. Surface sterilization for eliminating epiphytic microorganisms was performed according to the methods described by Mendes et al. (2007) with modifications to timing and concentrations. Briefly, fragments of each tissue were immersed separately in 70% ethanol for 3 min, 4% NaClO for 5 min, and 70% ethanol for 30 s; and finally, samples were rinsed in deionized water six times. The surface sterilization of tissues was confirmed by no growth of bacteria in Petri dishes with tryptic soy agar (TSA, BD, Difco, United States) inoculated with the last rinsing water and by negative PCR amplification of that same water.

### Metagenomic DNA Extraction, 16S rRNA Gene Amplification, and Pyrosequencing

We extracted DNA from 36 samples including three samples of root, phloem, and bark of each biological replicate from each pine species (Arizona and Durango pines). Tissues were independently ground to fine powder with sterilized mortars, pistils, and liquid nitrogen in a sterile environment. DNA of each sample was extracted using the protocol described by Zhou et al. (1996) with minor modifications. Briefly, 1 mL of lysis buffer (100 mM Tris-HCl pH 8, 100 mM EDTA pH 8, 100 mM sodium phosphate pH 8, 1.5 M NaCl, 1% CTAB, 1.2% Triton X-100) was added to ∼50 mg of each sample contained in 2 mL screw-cap tubes. Suspensions were vortexed for 10 min and 30 μL of lysozyme was added, after which samples were homogenized for 20 s and incubated at 37°C for 1 h. Proteins were degraded by the addition of 30 μL of proteinase K (10 mg/mL), vortexing for 10 s, and incubating at 60°C for 4 h with gentle end-over-end inversions every 30 min. The aqueous phase was collected after centrifugation at 14,000 × *g* for 2 min at room temperature (RT) into a new 2 mL sterile tube. Proteins were removed by the addition of an equal volume of phenol-chloroform-isoamyl alcohol (25:24:1, v/v/v) and the aqueous phase was recovered by centrifugation at 10,000 × *g* for 10 min (this step was repeated twice). The DNA was precipitated with the addition of an equal volume of cold isopropanol, overnight incubation at -20°C, and centrifugation at 10,000 × *g* for 20 min. The DNA pellet was washed twice with 250 μL cold ethanol at 10,000 × *g* for 5 min, and the ethanol was then discarded and the tube dried at RT, finally being resuspended in sterile deionized water. DNA was quantified using a NanoDrop 2000c Spectrophotometer (Thermo Scientific, Wilmington, DE, United States) and was observed in 1.0% agarose gel.

DNA from 36 samples was amplified independently with 8F and 556R primers (Navarro-Noya et al., 2013), tagged with 10 bp multiplex identifiers barcode (MID) and a Roche 454 adaptor (Roche, Mannheim, DE, United States) according to the Lib-L protocol. The V1-V3 region of the 16S rRNA gene was amplified as reported by Briones-Roblero et al. (2017a). Amplification products were purified using a QIAquick Gel Extraction kit (Qiagen, Valencia, CA, United States) according to the manufacturer’s protocol and quantified using a Nanodrop 2000c spectrophotometer. For pyrosequencing using a Roche GS-FLX Titanium 454 pyrosequencer (Roche, Mannheim, DE, United States) at Macrogen, Inc., (Seoul, South Korea), we pooled three amplification products from each tissue in equimolar concentrations (100 ng/μL), for a total of 12 libraries (**Table 1**).

**Table 1:** Sample ID and taxa number identified from phylum to genus.

Host	Tissue/biological replicate	Sample ID	Taxa number at level:				
			Phylum	Class	Order	Family	Genus
Durango pine	root1	RootPD1	4	7	9	15	16
	root2	RootPD2	7	11	16	23	26
	phloem1	PhloemPD1	5	9	14	20	18
	phloem2	PhloemPD2	7	9	10	14	11
	bark1	BarkPD1	6	13	24	38	42
	bark2	BarkPD2	4	7	12	17	12
Arizona pine	root1	RootPA1	6	12	17	25	25
	root2	RootPA2	5	10	16	25	21
	phloem1	PhloemPA1	6	9	13	20	16
	phloem2	PhloemPA2	2	5	9	13	10
	bark1	BarkPA1	6	11	20	32	36
	bark2	BarkPA2	–	–	–	–	–

### Analysis of Pyrosequenced Data

The raw sequencing data were analyzed using the Quantitative Insights Into Microbial Ecology (QIIME) pipeline v.1.8 (Caporaso et al., 2010). Low-quality reads were eliminated according to the follow filtration criteria: Phred quality score < 25, sequences < 150 or > 500 bp long, sequences that had homopolymers > 6, sequences that had any ambiguous characters, and any errors in barcodes.

High-quality sequences were sorted into operational taxonomic units (OTUs) at 97% of similarity threshold using the UCLUST algorithm (Edgar, 2010) based on the open-reference method. Chimeric sequences were detected and eliminated using Chimera Slayer (Haas et al., 2011). An OTU table was constructed with representative sequences for each OTU (longest) and their relative abundance across the samples. Sequences matching chloroplasts were manually removed. All representative sequences were aligned using PyNast (Caporaso et al., 2010) and the taxonomic assignment to the different levels, from phylum to genus, was performed at an 80% confidence threshold using the Ribosomal Database Project (RDP) Naïve Bayesian Classifier^1^ (Wang et al., 2007). In order to corroborate the taxonomic assignment of OTUs, they were manually compared against reference sequences deposited in three databases: RDP^2^, GenBank^3^ (Altschul et al., 1990), and Greengenes^4^ (DeSantis et al., 2006).

Samples were homogenized with respect to the sample with the lowest number of reads. The α- and β diversities were estimated in QIIME for each sample (Kuczynski et al., 2011). For estimation of α diversity, the replicates of each tissue of each pine species were averaged and used the following metrics: Chao1 (richness estimator), Shannon, Simpson, and Phylogenetic Diversity (PD) (diversity indices) (Magurran, 1998; Faith and Baker, 2007). Rarefaction curves and the Good’s coverage index were computed to determine sampling completeness (Chao et al., 1988). Owing to unequal variances, differences in the means of richness and diversity values were tested using Welch’s test (ANOVA).

A heatmap was constructed with the relative abundance information of bacterial genera identified and merged with a dendrogram built through the unweighted pair group method with arithmetic mean (UPGMA) method using the Bray–Curtis similarity index. The clusters reliability was supported by bootstrap test after 1000 pseudoreplicates and by the cophenetic correlation coefficient estimated in PAST v.3.11 (Hammer et al., 2001); the tree was visualized in iTol^5^ (Letunic and Bork, 2007).

Based on presence/absence data of bacterial genera, Venn diagrams were generated with the web application, Venny (Oliveros, 2007), for the following comparisons: (a) between the total community of pine species; (b) among tissue samples of each pine species; and (c) among the total community of each pine species and that previously reported for *D*. *rhizophagus* (Briones-Roblero et al., 2017a).

The β diversity of endophytic bacterial communities of each pine species was estimated using the Bray–Curtis dissimilarity index as well as unweighted and weighted Fast UniFrac distances metrics. Both UniFrac estimators are based on phylogenetic richness, but weighted UniFrac analysis also considers the relative abundance of OTUs (Lozupone et al., 2011). This information is extracted from a phylogenetic tree constructed with the maximum likelihood method in FastTree (Price et al., 2009), employing the generalized time-reversible (GTR) nucleotide model. A Monte Carlo test was applied to determine statistically significant differences in β-diversity values among bacterial communities after 1000 randomizations of the original UniFrac matrices. To compare endophytic bacterial communities among different tissues of Arizona and Durango pines, along with the gut community of *D. rhizophagus* (Briones-Roblero et al., 2017a), we carried out principal coordinate analyses (PCoA) using the Bray–Curtis dissimilarity index and both UniFrac (unweighted/weighted) indexes; the corresponding plots of these analyses were visualized in NTSYSpc v.2.02 (Rohlf, 1997). Analyses of similarity (ANOSIM) were conducted to establish significant differences in the community composition among the analyzed tissues, as well as between endophytic and gut bacterial communities.

To determine the degree of taxonomic relatedness (i.e., the relative closeness of species in the taxonomic hierarchy) of the most abundant members found in the gut of *D. rhizophagus* (*Acinetobacter*, *Rahnella, Serratia*, *Stenotrophomonas*, *Propionibacterium*, and *Pseudomonas*) and their equivalents in endophytic bacterial communities, we performed phylogenetic inference analyses of these bacteria. Sequences of bacterial communities from both pines and *D. rhizophagus* gut, along with certain reference sequences of the GenBank, including some of isolates of this insect, were aligned in Clustal X v.2.1 (Larkin et al., 2007) and trimmed at the 5′ and 3′ ends using SeaView v.4.4.2 (Gouy et al., 2010). The alignments were submitted to the PhyML Web server^6^ to select the most appropriate models of nucleotide evolution (Lefort et al., 2017) and perform maximum-likelihood phylogenetic analyses (Guindon et al., 2010). Bootstrap tests were conducted to determine clustering strength and trees generated were rooted with *Anabaena variabilis* as the outgroup. Lastly, trees were visualized in iTol^5^ (Letunic and Bork, 2007).

### Predictive Functional Profiling

The Phylogenetic Investigation of Communities by Reconstruction of Unobserved States (PICRUSt) software (Langille et al., 2013) was utilized through the web application Galaxy^7^ (Afgan et al., 2016) to investigate the predictive functional profile of bacterial communities of different tissues of each pine species. The sequences from each library were demultiplexed, and OTUs were clustered against the Greengenes database with the closed-reference method employing QIIME to generate an OTU table in biom-format. The accuracy of metagenome predictions was determined with the weighted nearest-sequenced taxon index (weighted NSTI). The NSTI summarizes the extent to which microorganisms in a sample are related to sequences genomes and represents the average branch length that separates each OTU in a sample from a reference bacterial genome, weighting their relative abundance in each sample. Low values of this index indicate a closer mean relationship. The table containing the predicted gene family-counts per sample, based on orthologous groups and identifiers following Kyoto Encyclopedia of Genes and Genomes (KEGG)^8^ at levels 2 and 3, was cleaned according to these criteria: removal of categories unrelated with bacterial physiology/metabolism (like human diseases) and removal of gene family categories with a count equal to 0. The database at level 2 was used to generate a heatmap in CIMminer^9^ and it was merged with a dendrogram built through UPGMA method using the Gower similarity index. Tree reliability was supported by a bootstrap test after 1000 pseudoreplicates and by the cophenetic correlation coefficient estimated in PAST v.3.11 (Hammer et al., 2001). The database at level 3 was employed to build a bar chart with the relative abundance information of the metabolic pathways. Spearman’s correlation was performed to associate the abundances of inferred metabolic functions of pine endophytes and *D. rhizophagus* gut communities obtained in PICRUSt, at the level 2 of metabolism category, with PAST v.3.11 (Hammer et al., 2001).

### Data Accessibility

The Standard Flowgram Format (SFF) file was deposited in the NCBI SRA database under bioproject PRJNA395769 with biosample SAMN07414558 and accession number SRR5872379.

## Results

### Pyrosequencing Data

A total of 529 bacterial OTUs were identified from 23,330 high-quality and non-plastid sequences in 11 of 12 samples. A biological replicate corresponding to the bark of Arizona pine was discarded given that it presented an insufficient number of reads. The remaining 11 samples featured an average length of 335 bp/read, and an average of 2,120 reads/sample (**Table 1**). The bark of both pine species exhibited the highest OTUs number, and meanwhile, phloem had the lowest OTUs numbers (**Table 2**).

**Table 2:** Richness and α diversity indices for root, phloem, and bark samples of Durango and Arizona pines.

Host	Tissue	Good’s coverage (%)^a^	Observed OTUs^97%,a^	Chao1^a^	Simpson^a^	Shannon^a^	PD^a,b^
Durango pine	Root	94	25	116	0.56	2.1	1.77
	Phloem	96	20	46	0.48	1.79	1.21
	Bark	63	144	529	0.96	6.25	9.38
Arizona pine	Root	93	29	111	0.58	2.25	2.56
	Phloem	94.5	21	104	0.48	1.77	1.11
	Bark	74	97	443	0.91	4.89	7.17

^a^*Mean values between two biological replicates for each tissue, except in bark of Arizona pine. ^b^Phylogenetic diversity index*.

### Bacterial Relative Abundance

**FIGURE 1:**
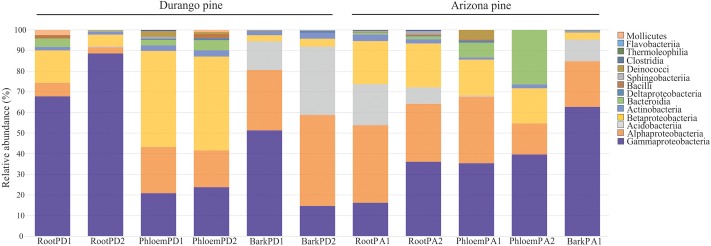
Distribution of relative abundance of endophytic bacterial associated with root, phloem, and bark of Durango and Arizona pines at the class level (RootPD1 = Root *P. durangensis*1, RootPD2 = Root *P. durangensis*2, PhloemPD1 = Phloem *P. durangensis*1, PhloemPD2 = Phloem *P. durangensis*2, BarkPD1 = Bark *P. durangensis*1, BarkPD2 = Bark *P. durangensis*2; RootPA1 = Root *P. arizonica*1, RootPA2 = Root *P. arizonica*2, PhloemPA1 = Phloem *P. arizonica*1, PhloemPA2 = Phloem *P. arizonica*2, BarkPA1 = Bark *P. arizonica*1).

A total of seven phyla, 14 classes, 26 orders, 43 families, and 51 bacterial genera were identified in roots, phloem, and bark of Arizona and Durango pines samples. The taxa number from phylum to family were not the same among tissues and between pine species (**Table 1**), their frequencies varied slightly between replicates of the same tissue, among tissues of the same pine species, and between pines species. In particular, Proteobacteria was the most abundant phylum in all samples, followed by Acidobacteria, Actinobacteria, Firmicutes, Bacteroidetes, Thermi, Tenericutes, and several taxa unassigned to a phylum (Supplementary Figure S1A); among classes, Gammaproteobacteria was the most dominant, followed by Alphaproteobacteria, Acidobacteriia, Betaproteobacteria, Actinobacteria, and other in low frequencies (**Figure 1**); and finally, among families, Enterobacteriaceae together with Acetobacteraceae and Acidobacteriaceae represented ∼80% total reads (Supplementary Figure S1B).

**FIGURE 2:**
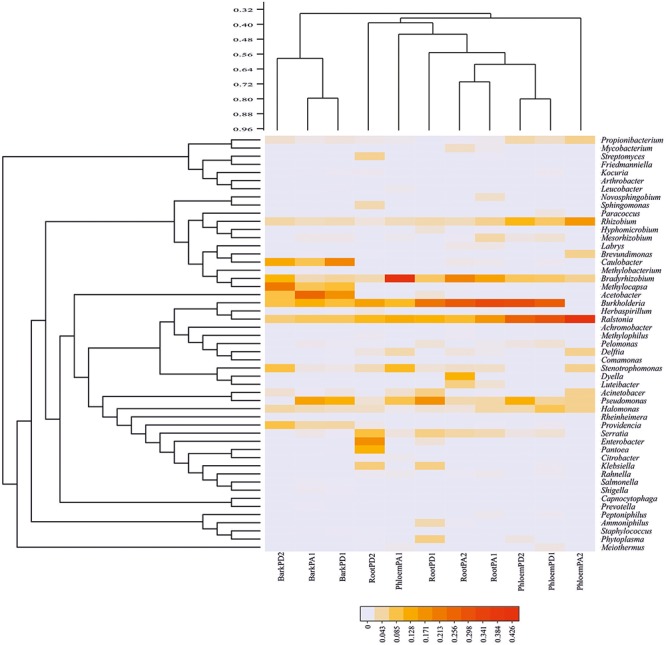
Heatmap of relative abundance of endophytic bacterial genera in Durango and Arizona pines. Bacterial community distribution of the 51 bacterial genera among root, phloem, and bark samples (cophenetic correlation = 0.90) (RootPD1 = Root *P. durangensis*1, RootPD2 = Root *P. durangensis*2, PhloemPD1 = Phloem *P. durangensis*1, PhloemPD2 = Phloem *P. durangensis*2, BarkPD1 = Bark *P. durangensis*1, BarkPD2 = Bark *P. durangensis*2; RootPA1 = Root *P. arizonica*1, RootPA2 = Root *P. arizonica*2, PhloemPA1 = Phloem *P. arizonica*1, PhloemPA2 = Phloem *P. arizonica*2, BarkPA1 = Bark *P. arizonica*1).

A total 51 bacterial genera were recovered combining manual and QIIME assignment, among which *Acetobacter* (14.20%), *Burkholderia* (13.55%), *Caulobacter* (11.15%), *Pseudomonas* (10.27%), *Ralstonia* (10.03%), *Bradyrhizobium* (7.00%), and *Methylocapsa* (6.67%) were the best represented genera, followed by *Rhizobium* (3.64%), *Providencia* (3.33%), *Halomonas* (3.03%), *Stenotrophomonas* (1.76%), *Serratia* (1.74%), *Propionibacterium* (1.36%), *Enterobacter* (1.29%), and *Mesorhizobium* (1.10%); the 36 remaining genera exhibited frequencies < 1.00% of total reads (**Figure 2** and Supplementary Table S1).

### α- and β-Diversity Analysis

Overall, the Good’s coverage suggested that the sampling effort for bark samples of Durango and Arizona pines was acceptable (>63%), and for phloem and root samples it was appropriate (>93%), indicating that the current sampling effort was adequate to obtain the most abundant OTUs (**Table 2**). Rarefaction curves exhibited similar results, and these tended toward saturation in the phloem and root samples, but not in bark samples, where more sampling effort is necessary (Supplementary Figure S2). No significant differences were observed in bacterial richness and diversity among tissue samples of both pine species with Chao1 (Welch *F*-test: *F* = 4.59, *p* = 0.169) and Shannon index (Welch *F*-test: *F* = 10.62, *p* = 0.067), respectively. In contrast, the phylogenetic diversity with PD index (Welch *F*-test: *F* = 29.41, *p* = 0.026) and the dominance estimated with the Simpson index (Welch *F*-test: *F* = 26.75, *p* = 0.020) were statistically significant, suggesting that bark samples of both pine species were different to roots and phloem samples (**Table 2**).

**FIGURE 3:**
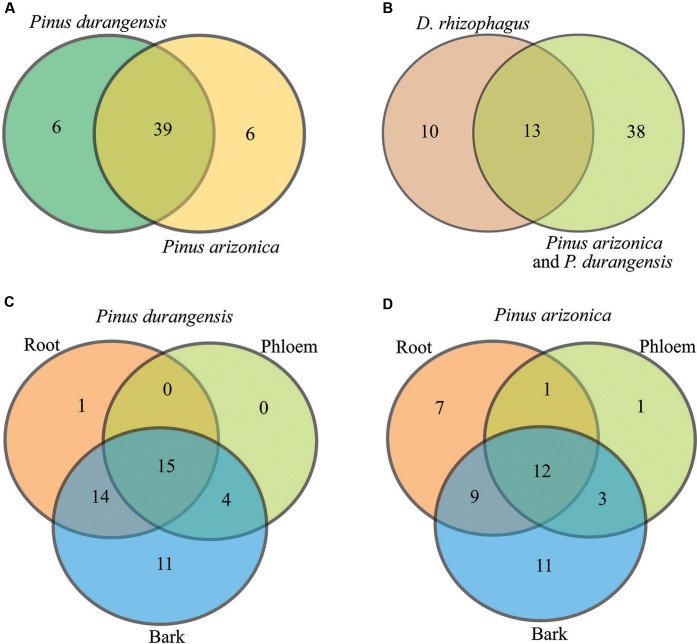
Venn diagrams showing shared and unique genera. **(A)** *P. durangensis* vs. *P. arizonica*, **(B)** *D. rhizophagus* vs. *P. arizonica* and *P. durangensis*, **(C)** *P. durangensis* (root vs. phloem vs. bark), and **(D)** *P. arizonica* (root vs. phloem vs. bark).

**FIGURE 4:**
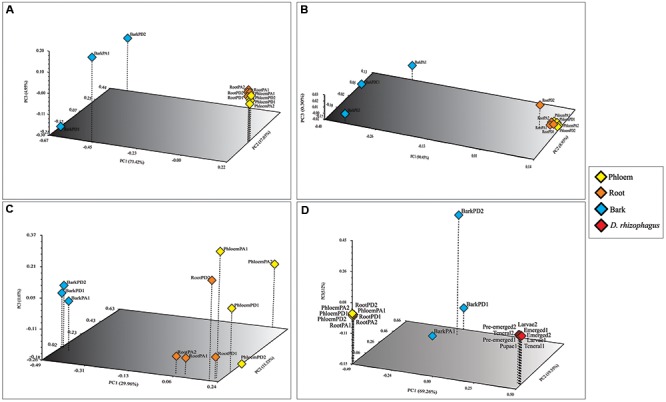
Principal coordinate analyses (PCoA) of the endophytic bacterial communities associated with root, phloem, and bark of Durango and Arizona pines samples. **(A)** Bray–Curtis dissimilarity, **(B)** weighted and, **(C)** unweighted UniFrac distances; and PCoA of the endophytic bacterial communities associated with root, phloem, and bark of Durango and Arizona pines and *D. rhizophagus* samples **(D)** Bray–Curtis dissimilarity. The percentages of variation explained by each axis are shown in parentheses. Orange diamonds represent the root samples. Yellow diamonds represent the phloem samples. Blue diamonds represent the bark samples. Red diamonds represent the *D. rhizophagus* samples (RootPD1 = Root *P. durangensis*1, RootPD2 = Root *P. durangensis*2, PhloemPD1 = Phloem *P. durangensis*1, PhloemPD2 = Phloem *P. durangensis*2, BarkPD1 = Bark *P. durangensis*1, BarkPD2 = Bark *P. durangensis*2; RootPA1 = Root *P. arizonica*1, RootPA2 = Root *P. arizonica*2, PhloemPA1 = Phloem *P. arizonica*1, PhloemPA2 = Phloem *P. arizonica*2, BarkPA1 = Bark *P. arizonica*1).

The bacterial communities were very similar between pine species, sharing 39 of the 51 bacterial genera identified (**Figure 3A**). Among root, phloem, and bark samples of Durango pine, 15 bacterial genera were shared, root and bark samples shared 14 genera, and four genera were present in bark and phloem samples; 11 and one bacterial genera were exclusive of bark and root, respectively, in this pine species (**Figure 3C**). In the case of Arizona pine, 12 genera were shared among roots, phloem, and bark samples, nine genera were shared exclusively between root and bark samples, three were shared between bark and phloem samples, and only one was shared between root and phloem samples; seven were found to be unique to root, one to phloem, and 11 to bark (**Figure 3D**). Finally, the comparison between pine species and gut bacteria of the bark beetle *D. rhizophagus* demonstrated that 13 genera were shared by these pines and the insect, 10 were exclusive of *D. rhizophagus*, and 38 exclusive to both pine species (**Figure 3B**; detailed information on bacterial genera available in Supplementary Table S2). The three first coordinates using the Bray–Curtis dissimilarity (**Figure 4A**), weighted UniFrac (**Figure 4B**), and unweighted UniFrac (**Figure 4C**) estimators explained 95.4, 99.68, and 56.57% of the total observed variation in each analysis, respectively. The PCoA showed significant differences between tissue samples (Bray–Curtis ANOSIM, *p* = 0.03; weighted UniFrac ANOSIM, *p* = 0.03; and unweighted UniFrac ANOSIM, *p* = 0.02), revealing that bark bacterial communities of both pines species were very similar, but different markedly with respect to those in phloem and root (**Figures 4A–C** and **Table 2**). These two last bacterial communities were similar in relative abundance, though varied in terms of richness. In the case of bacterial communities of Arizona and Durango pines and those previously reported of *D. rhizophagus* gut, the first three coordinates explained 93.93% of the total variation (**Figure 4D**). The PCoA showed an evident spatial segregation of communities of pine tissues and *D. rhizophagus* gut, with differences that were statistically significant (Bray–Curtis ANOSIM, *p* = 0.001). Phylogenetic inference analyses indicated there to be a high degree of taxonomic relatedness between the most abundant bacteria in the *D. rhizophagus* gut and their equivalent in endophytic communities (Supplementary Figure S3), that is all sequences constituted monophyletic groups at the genus level. In some cases, the topologies suggested a high taxonomic closeness at the species level (e.g., *Rahnella* and *Pseudomonas*), but others (e.g., *Acinetobacter*, *Propionibacterium*, *Serratia*, and *Stenotrophomonas*) suggested the presence of different species.

### Functional Profiling Prediction

**FIGURE 5:**
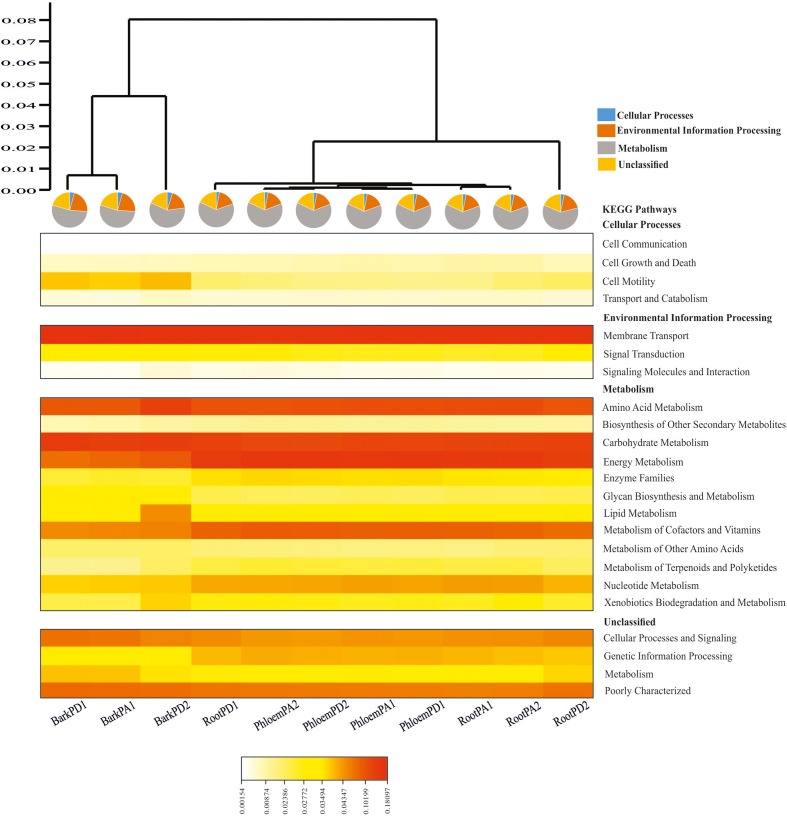
Heatmap depicting the PICRUSt-inferred gene relative abundance in the predicted endophytic bacterial communities of root, phloem, and bark of Arizona and Durango pines samples (cophenetic correlation = 0.98). Warm colors represent high abundances and clear colors represent low abundances (RootPD1 = Root *P. durangensis*1, RootPD2 = Root *P. durangensis*2, PhloemPD1 = Phloem *P. durangensis*1, PhloemPD2 = Phloem *P. durangensis*2, BarkPD1 = Bark *P. durangensis*1, BarkPD2 = Bark *P. durangensis*2; RootPA1 = Root *P. arizonica*1, RootPA2 = Root *P. arizonica*2, PhloemPA1 = Phloem *P. arizonica*1, PhloemPA2 = Phloem *P. arizonica*2, BarkPA1 = Bark *P. arizonica*1).

The PICRUSt analysis yielded average weighted NSTI values of 0.24 ± 0.07_sd_ and 0.20 ± 0.09_sd_ for Arizona and Durango pines samples, indicating that functional gene predictions were accurate in all samples. Among the predicted KEGG pathways at level 2, the most significant were those related to membrane transport (14.56%), energy metabolism (11.59%), carbohydrate metabolism (10.66%), amino acid metabolism (10.18%), and metabolism of cofactors and vitamins (6.68%) in terms of total pathways (**Figure 5**). The metabolic pathways at level 3, which considered metabolic capabilities more specific, were: oxidative phosphorylation and methane metabolism (energy metabolism); glycolysis-gluconeogenesis, and metabolism of different substrates, such as starch, sucrose, pyruvate, amino sugar, and nucleotide sugars (carbohydrate metabolism); terpenoid backbone biosynthesis and prenyltransferases (metabolism of terpenoids and polyketides); glutathione metabolism, ubiquinone and other terpenoid-quinone biosynthesis, pantothenate and CoA biosynthesis (metabolism of cofactors and vitamins) (Supplementary Figure S4). The Spearman correlation of metabolic profiles showed there to be a strong positive association between endophytic and gut bacterial communities (*r_s_* = 0.94; *p* < 0.05; Supplementary Table S3).

## Discussion

In the present study, the endophytic bacterial community in roots, phloem, and bark of healthy saplings of Arizona and Durango pines was analyzed with pyrosequencing 454. Overall, Enterobacteriaceae, Acetobacteraceae, and Acidobacteriaceae were the endophytic dominant families. Previous studies using NGS technologies have demonstrated the dominance of these families (and their corresponding phyla) in needles of other mature coniferous trees, e.g., *Picea engelmannii*, *Pinus flexilis*, *P*. *contorta*, and *P*. *radiata* (Carrell and Frank, 2014; Carrell et al., 2016; Rúa et al., 2016). This information suggests that dominance of these taxa in conifers is apparently independent of the host species and age, type of tissue, geographic location, or sample processing (i.e., surface sterilization, DNA extraction protocol). Several constrains or selective factors may be responsible for this asymmetry in favor of these families, including the tree inner-environment, local interactions among different bacterial groups, bacterial metabolic capacities, and the historical association between these phyla and conifers (Hardoim et al., 2015).

At the genus level, a total of 51 bacterial genera were identified in both pine species, from which 39 genera were shared, six were exclusive to Arizona pine, and six exclusive to Durango pine. Previous studies using both culture-dependent and culture-independent techniques (e.g., DGGE, molecular cloning) have reported from one to eight bacterial genera, among which are *Bacillus*, *Brevibacillus*, *Brevundimonas*, *Burkholderia*, *Cellulomonas*, *Kocuria*, *Methylobacterium*, *Paenibacillus*, *Pseudomonas*, and *Rahnella* (Shishido et al., 1995; Pirttilä et al., 2000; Cankar et al., 2005; Izumi et al., 2008; Bal et al., 2012), representing <10% of the bacterial genera recovered in this study using pyrosequencing. These differences reflect, as it is known, the limited statistical coverage of conventional methods; however, traditional culture methods remain necessary for obtaining more detailed information about functional capacities.

The comparison between both pine species at the genus level exhibits that bacterial communities are similar in presence and abundance of taxa. It is difficult to establish a comparison at this taxonomic category with previous studies using NGS technologies, because most of them have not reported this information (Carrell et al., 2016) or there is only partial information available (Carrell and Frank, 2014, 2015; Rúa et al., 2016). However, we assume that scarce differences observed may be because of several factors as host–plant genotype, plant organs, vegetative stage, soil type, and environmental stress as was described for agricultural crops where structuring of the endophytic bacteria communities has been evident (Sturz et al., 1998; Reiter et al., 2002; Sessitsch et al., 2002; Conn and Franco, 2004).

Most of the dominant bacterial genera identified in this study (e.g., *Bradyrhizobium, Burkholderia*, *Pseudomonas*, *Ralstonia*, and *Rhizobium*) were present in all plant tissues of both pines species, whereas certain genera with frequencies <1.0% were exclusive to a particular tissue. This result indicates the lack of structuring of the dominant members of the community at genera level with respect to a specific tissue, which is the opposite of what was reported in other works (e.g., Sun et al., 2008; Reinhold-Hurek and Hurek, 2011; Garcias-Bonet et al., 2012; Xia et al., 2015; Liu et al., 2016). Whereas the tissue specificity may be explained by differences with regards to range of physical and chemical environmental conditions, such as light, oxygen concentration gradient, and toxic metabolites present in each tissue (Garcias-Bonet et al., 2012), the non-structuring may only be possible assuming that these factors in the inner-environment of conifers are similar or homogeneous in different tissues, which, although it has not been evaluated, seems to be highly unlikely. Another possible explication to be considered is that the dominant members have metabolic capacities that allow them to face and tolerate heterogeneous inner-environments and restrain community members that do not have these capacities in specific tissues. Beyond these assumptions, the structuring of these communities could be observed at finer taxonomic levels (i.e., species or strain) than those resolved by the V1–V3 16S regions in this study.

The α diversity of bacterial communities in both pine species was higher in bark than in other tissues. This higher diversity could be understood via several factors, including horizontal transmission by airborne contamination (Whipps et al., 2008), raindrops (Morris, 2002), and dissemination between plants (Vandenkoornhuyse et al., 2015). In addition, the bark is rich in organic nutrients, resulting in it being a target for many different organisms (e.g., insects, vertebrates, fungi, bacteria) (Franceschi et al., 2005), particularly in the early years of cambium activity. Regarding the lower diversity observed in roots, this might be because most coniferous species grow in suboptimal soils and climates (Moyes et al., 2016); in fact, higher diversity values have been determined in roots versus other tissues in crop plants growing in soils with greater quality and nutritional content (Albareda et al., 2006; Zarraonaindia et al., 2015). In the case of phloem in pines, diversity values obtained using culture-dependent techniques have been very low (<10 colony-forming units) (Mason et al., 2015), and to our knowledge, there are no reports of using culture-independent methods.

The PCoA analyses employing all metrics clearly separate bacterial communities of bark samples from those in root and phloem in both pine species. These differences are derived by some of the most abundant genera (*Acetobacter* and *Methylocapsa*) in bark and bacteria present or absent (*Methylobacterium*, *Kocuria*, *Arthrobacter*, *Staphylococcus*, *Paracoccus*, *Comamonas*, *Prevotella*, *Salmonella*, *Shigella*, *Sphingomonas*, *Rheinheimera*, *Achromobacter*, *Capnocytophaga*, *Leucobacter*, and *Friedmaniella*) in the different communities and with low abundance (<1.0% reads) (*p* < 0.05). Unfortunately, it is difficult to determine whether these differences are consistent among other pine or coniferous species because, until this work, there have been no other studies evaluating endospheric tissues in roots, phloem, and bark using NGS technologies. Additionally, owing to the low number of biological replicates in this study, caution in interpreting inferences about the presence/absence of particular endophytic genera in the tissues of pines is prudent.

The comparison between communities of pines and *D. rhizophagus* gut shows statistically significant differences (*p* < 0.05); however, several of the endophytic bacterial genera identified in this study apparently have taxonomic relatedness with the most abundant members of the core bacteriome of this bark beetle (Briones-Roblero et al., 2017a). While the communities of Arizona and Durango pines are more diverse in a 2:1 ratio than those previously found in *D. rhizophagus*, 13 bacterial genera of this insect are shared with hosts. An aspect that should be highlighted is that among shared genera, the most abundant in pines are not necessarily the most abundant in the insect gut, and vice versa, which might be a consequence of the different environmental conditions where bacteria develop (endosphere and gut). Several of these shared genera (e.g., *Pseudomonas*, *Serratia*, *Rahnella*) have also been found in subcortical galleries and surrounding uninfested tissues of trees colonized by other *Dendroctonus* bark beetles (Durand et al., 2015; Mason et al., 2015). Likewise, other shared taxa (e.g., *Acinetobacter*, *Burkholderia, Enterobacter*, *Kocuria*, *Methylobacterium*, *Pantoea, Prevotella, Propionibacterium*, *Providencia*, *Pseudomonas*, and *Stenotrophomonas*) have been reported as endophytes of pines and in numerous tissues of plants (Pirttilä et al., 2000; Strzelczyk and Li, 2000; Bal et al., 2012; Hardoim et al., 2015; Kõiv et al., 2015; Moyes et al., 2016; Yang et al., 2017).

Although it is unknown how this bark beetle acquires their symbiotic bacteria, the degree of taxonomic relatedness of bacterial genera between both assemblages suggests that at least some gut bacteria might be associated with those from pine tissues. In fact, phylogenies of the dominant bacterial genera in the gut, their equivalent endophytes, and other reference sequences of these same bacterial genera of the GenBank, formed robust monophyletic groups (boostrap value > 50%). Similarly, Hernández-García et al. (2017) found a lack of phylogenetic consistence between *Dendroctonus* bark beetles and their bacterial assemblages, suggesting that bacteria might be environmentally acquired. While the horizontal transmission of endophytic communities has been demonstrated in other hemimetabolous insects that feed on phloem (Lòpez-Fernàndez et al., 2017), this has not been tested in holometabolous insects as bark beetles. Specific experimental studies under controlled laboratory conditions are necessary to determine this apparent horizontal transmission in bark beetles.

Lastly, the positive association found between the metabolism of endophytic and bark beetle gut bacterial communities suggests that certain predicted functional pathways are shared by both communities (e.g., amino acids, carbohydrates, biosynthesis of secondary metabolites, energy and cofactors, vitamins metabolism, xenobiotic biodegradation). Some of these metabolic routes have also been reported in bacterial communities associated with other phloem- and wood-feeding insects as well as other *Dendroctonus* species (Supplementary Figure S5) using PICRUSt or shotgun metagenomics (Scully et al., 2013; Berasategui et al., 2016; Hernandez-García et al., unpublished data). In addition, certain particular metabolic functions (e.g., hydrolysis of lipids, starch, esters, xylan, and cellulose, as well as xenobiotic biodegradation) have been demonstrated in bacteria isolated from the gut of particular *Dendroctonus* bark beetles (Morales-Jiménez et al., 2012, 2013; Hu et al., 2014; Cano-Ramírez et al., 2016; Briones-Roblero et al., 2017b). Likewise, several of these metabolic pathways (oxidative phosphorylation, xenobiotic degradation, methane, glutathione, amino sugar and nucleotide sugar metabolism, and carbohydrates metabolism) have been described in endophytic bacterial communities of many plants using PICRUSt, and in some cases confirmed with bacterial isolates (Van Aken et al., 2004; Parmentier et al., 2011; Sheibani-Tezerji et al., 2015; Ruiz-Pérez et al., 2016; Sánchez-López et al., 2017).

In brief, this is the first study that investigates the diversity and structure of the endophytic bacterial community in roots, phloem, and bark of Arizona and Durango pines. Our findings show there to be a bacterial community similar to those reported for other pine species and coniferous genera. Our results also demonstrate that few dominant members of these communities are shared among different types of tissues and some low-abundance taxa are exclusive to particular tissues. Several genera of this endophytic community showed there to be taxonomic relatedness with gut-dominant members of the bark beetle, *D. rhizophagus*, suggesting that some could be the same species. However, further evidence is necessary to clarify if they are the same species or not, and corroborate their mode of transmission. Lastly, our results indicate there is a strong metabolic association at the community level, although large differences in ecological and functional traits could be observed at the species level within the same genera or families, and as a consequence, they can display different ecological responses depending on the habitat or environment. Future research based on “omics” technologies (e.g., genomics, transcriptomics, proteomics, metabolomics), as well as the assessment of metabolic capacities in microorganisms isolated from these pines will be needed to reach a better understanding of the pine-bacteria relationship.

## Author Contributions

Conception of the work: RG-E, FR-O, and GZ; funding acquisition: FR-O and GZ; analysis and interpretation of data for the work: RG-E, CB-R, RP-M, FR-O, and GZ; drafting the work: RG-E, FR-O, and GZ; revising it critically for important intellectual content: RG-E, CB-R, FR-O, and GZ. All authors approved the manuscript.

## Conflict of Interest Statement

The authors declare that the research was conducted in the absence of any commercial or financial relationships that could be construed as a potential conflict of interest.

## Footnotes

1 http://rdp.cme.msu.edu/classifier/classifier.jsp

2 http://rdp.cme.msu.edu/seqmatch/seqmatch_intro.jsp

3 http://blast.ncbi.nlm.nih.gov/Blast.cgi

4 http://greengenes.lbl.gov/

5 http://itol.embl.de/

6 http://www.atgc-montpellier.fr/phyml/

7 https://huttenhower.sph.harvard.edu/galaxy/

8 www.genome.jp/kegg/

9 http://discover.nci.nih.gov

## References

[B1] Adams A. S. Aylward F. O. Adams S. M. Erbilgin N. Aukema B. H. Currie C. R. (2013). Mountain pine beetles colonizing historical and naïve host trees are associated with a bacterial community highly enriched in genes contributing to terpene metabolism. *Appl. Environ. Microbiol.* 79 3468–3475. 10.1128/AEM.00068-13 23542624 PMC3648045

[B2] Adams A. S. Currie C. R. Cardoza Y. Klepzig K. D. Raffa K. F. (2009). Effects of symbiotic bacteria and tree chemistry on the growth and reproduction of bark beetle fungal symbionts. *Can. J. For. Res.* 39 1133–1147. 10.1139/X09-034

[B3] Afgan E. Baker D. van den Beek M. Blankenberg D. Bouvier D. Čech M. (2016). The Galaxy platform for accessible, reproducible and collaborative biomedical analyses: 2016 update. *Nucleic Acids Res.* 44 W3–W10. 10.1093/nar/gkw343 27137889 PMC4987906

[B4] Albareda M. Dardanelli M. S. Sousa C. Megías M. Temprano F. Rodríguez-Navarro D. N. (2006). Factors affecting the attachment of rhizospheric bacteria to bean and soybean roots. *FEMS Microbiol. Lett.* 259 67–73. 10.1111/j.1574-6968.2006.00244.x 16684104

[B5] Altschul S. F. Gish W. Miller W. Myers E. W. Lipman D. J. (1990). Basic local alignment search tool. *J. Mol. Biol.* 215 403–410. 10.1016/S0022-2836(05)80360-2 2231712

[B6] Bal A. Anand R. Berge O. Chanway C. P. (2012). Isolation and identification of diazotrophic bacteria from internal tissues of *Pinus contorta* and *Thuja plicata*. *Can. J. For. Res.* 42 807–813. 10.1139/x2012-023

[B7] Berasategui A. Axelsson K. Nordlander G. Schmidt A. Borg-Karlson A. K. Gershenzon J. (2016). The gut microbiota of the pine weevil is similar across Europe and resembles that of other conifer-feeding beetles. *Mol. Ecol.* 25 4014–4031. 10.1111/mec.13702 27199034

[B8] Boone C. K. Keefover-Ring K. Mapes A. C. Adams A. S. Bohlmann J. Raffa K. F. (2013). Bacteria associated with a tree-killing insect reduce concentrations of plant defense compounds. *J. Chem. Ecol.* 39 1003–1006. 10.1007/s10886-013-0313-0 23807433

[B9] Briones-Roblero C. I. Hernández-García J. A. Gonzalez-Escobedo R. Soto-Robles L. V. Rivera-Orduña F. N. Zúñiga G. (2017a). Structure and dynamics of the gut bacterial microbiota of the bark beetle, *Dendroctonus rhizophagus* (Curculionidae: Scolytinae) across their life stages. *PLOS ONE* 12:e0175470. 10.1371/journal.pone.0175470 28406998 PMC5391025

[B10] Briones-Roblero C. I. Rodríguez-Díaz R. Santiago-Cruz J. A. Zúñiga G. Rivera-Orduña F. N. (2017b). Degradation capacities of bacteria and yeasts isolated from the gut of *Dendroctonus rhizophagus* (Curculionidae: Scolytinae). *Folia Microbiol.* 62 1–9. 10.1007/s12223-016-0469-4 27544667

[B11] Cankar K. Kraigher H. Ravnikar M. Rupnik M. (2005). Bacterial endophytes from seeds of Norway spruce (*Picea abies* L. Karst). *FEMS Microbiol. Lett.* 244 341–345. 10.1016/j.femsle.2005.02.008 15766788

[B12] Cano-Ramírez C. Santiago-Hernández A. Rivera-Orduña F. N. García-Huante Y. Zúñiga G. Hidalgo-Lara M. E. (2016). Expression, purification and characterization of an endoglucanase from *Serratia proteamaculans* CDBB-1961, isolated from the gut of *Dendroctonus adjunctus* (Coleoptera: Scolytinae). *AMB Express* 6:63. 10.1186/s13568-016-0233-9 27576896 PMC5005244

[B13] Caporaso J. G. Kuczynski J. Stombaugh J. Bittinger K. Bushman F. D. Costello E. K. (2010). QIIME allows analysis of high- throughput community sequencing data. *Nat. Methods* 7 335–336. 10.1038/nmeth.f.303 20383131 PMC3156573

[B14] Cardoza Y. J. Klepzig K. D. Raffa K. F. (2006). Bacteria in oral secretions of an endophytic insect inhibit antagonistic fungi. *Ecol. Entomol.* 31 636–645. 10.1111/j.1365-2311.2006.00829.x

[B15] Carrell A. A. Carper D. L. Frank A. C. (2016). Subalpine conifers in different geographical locations host highly similar foliar bacterial endophyte communities. *FEMS Microbiol. Ecol.* 92:fiw124. 10.1093/femsec/fiw124 27267931

[B16] Carrell A. A. Frank A. C. (2014). *Pinus flexilis* and *Picea engelmannii* share a simple and consistent needle endophyte microbiota with a potential role in nitrogen fixation. *Front. Microbiol.* 5:333 10.3389/fmicb.2014.00333 PMC4082182 25071746

[B17] Carrell A. A. Frank A. C. (2015). Bacterial endophyte communities in the foliage of coast redwood and giant sequoia. *Front. Microbiol.* 6:e01008. 10.3389/fmicb.2015.01008 26441933 PMC4585279

[B18] Chao A. Lee S.-M. Chen T. C. (1988). A generalized Good’s nonparametric coverage estimator. *Chin. J. Math.* 16 189–199.

[B19] Conn V. Franco C. (2004). Analysis of the endophytic actinobacterial population in the roots of wheat (*Triticum aestivum* L.) by terminal restriction fragment length polymorphism and sequencing of 16S rRNA clones analysis of the endophytic actinobacterial population in the roots. *Appl. Environ. Microbiol.* 70 1787–1794. 10.1128/AEM.70.3.1787-1794.2004 15006805 PMC368409

[B20] DeSantis T. Z. Hugenholtz P. Larsen N. Rojas M. Brodie E. L. Keller K. (2006). Greengenes, a chimera-checked 16S rRNA gene database and workbench compatible with ARB. *Appl. Environ. Microbiol.* 72 5069–5072. 10.1128/AEM.03006-05 16820507 PMC1489311

[B21] Dohet L. Grégoire J. C. Berasategui A. Kaltenpoth M. Biedermann P. H. (2016). Bacterial and fungal symbionts of parasitic Dendroctonus bark beetles. *FEMS Microbiol. Ecol.* 92:fiw129. 10.1093/femsec/fiw129 27387908

[B22] Durand A. A. Bergeron A. Constant P. Buffet J. P. Déziel E. Guertin C. (2015). Surveying the endomicrobiome and ectomicrobiome of bark beetles: the case of Dendroctonus simplex. *Sci. Rep.* 5:17190. 10.1038/srep17190 26608752 PMC4660424

[B23] Edgar R. C. (2010). Search and clustering orders of magnitude faster than BLAST. *Bioinformatics* 26 2460–2461. 10.1093/bioinformatics/btq461 20709691

[B24] Faith D. P. Baker A. M. (2007). Phylogenetic diversity (PD) and biodiversity conservation: some bioinformatics challenges. *Evol. Bioinform.* 2 121–128. 10.4137/EBO.S0 19455206 PMC2674678

[B25] Franceschi V. R. Krokene P. Christiansen E. Krekling T. (2005). Anatomical and chemical defenses of confer bark against bark beetles and other pests. *New Phytol.* 167 353–375. 10.1111/j.1469-8137.2005.01436.x 15998390

[B26] Garcias-Bonet N. Arrieta J. M. de Santana C. N. Duarte C. M. Marbà N. (2012). Endophytic bacterial community of a Mediterranean marine angiosperm (*Posidonia oceanica*). *Front. Microbiol.* 3:342. 10.3389/fmicb.2012.00342 23049528 PMC3448135

[B27] Gouy M. Guindon S. Gascuel O. (2010). SeaView version 4: a multiplatform graphical user interface for sequence alignment and phylogenetic tree building. *Mol. Biol. Evol.* 27 221–224. 10.1093/molbev/msp259 19854763

[B28] Gray C. A. Runyon J. B. Jenkins M. J. Giunta A. D. (2015). Mountain pine beetles use volatile cues to locate host limber pine and avoid non-host great basin bristlecone pine. *PLOS ONE* 10:e0135752. 10.1371/journal.pone.0135752 26332317 PMC4558103

[B29] Guindon S. Dufayard J. F. Lefort V. Anisimova M. Hordijk W. Gascuel O. (2010). New algorithms and methods to estimate maximum-likelihood phylogenies: assessing the performance of PhyML 3.0. *Syst. Biol.* 59 307–321. 10.1093/sysbio/syq010 20525638

[B30] Haas B. J. Gevers D. Earl A. M. Feldgarden M. Ward D. V. Giannoukos G. (2011). Chimeric 16S rRNA sequence formation and detection in Sanger and 454-pyrosequenced PCR amplicons. *Genome Res.* 21 494–504. 10.1101/gr.112730.110 21212162 PMC3044863

[B31] Hallmann J. Quadt-Hallmann A. Mahaffee W. F. Kloepper J. W. (1997). Bacterial endophytes in agricultural crops. *Can. J. Microbiol.* 43 895–914. 10.1139/m97-131

[B32] Hammer Ø. Harper D. A. T. Ryan P. D. (2001). PAST: paleontological statistics software package for education and data analysis. *Palaeontol. Electron.* 4 1–9.

[B33] Hardoim P. van Overbeek L. Berg G. Pirttilä A. M. Compant S. Campisano A. (2015). The hidden world within plants: ecological and evolutionary considerations for defining functioning of microbial endophytes. *Microbiol. Mol. Biol. Rev.* 79 293–320. 10.1128/MMBR.00050-14 26136581 PMC4488371

[B34] Hashem A. Abd-Allah E. F. Alqarawi A. A. Al-Huqail A. A. Wirth S. Egamberdieva D. (2016). The interaction between arbuscular mycorrhizal fungi and endophytic bacteria enhances plant growth of *Acacia gerrardii* under salt stress. *Front. Microbiol.* 7:1089. 10.3389/fmicb.2016.01089 27486442 PMC4949997

[B35] Hernández-García J. A. Briones-Roblero C. I. Rivera-Orduña F. N. Zúñiga G. (2017). Revealing the gut bacteriome of Dendroctonus bark beetles (Curculionidae: Scolytinae): diversity, core members and co-evolutionary patterns. *Sci. Rep.* 7:13864. 10.1038/s41598-017-14031-6 29066751 PMC5655642

[B36] Hu X. Yu J. Wang C. Chen H. (2014). Cellulolytic bacteria associated with the gut of *Dendroctonus armandi* larvae (Coleoptera: Curculionidae: Scolytinae). *Forests* 5 455–465. 10.3390/f5030455

[B37] Izumi H. Anderson I. C. Killham K. Moore E. R. (2008). Diversity of predominant endophytic bacteria in European deciduous and coniferous trees. *Can. J. Microbiol.* 54 173–179. 10.1139/W07-134 18388988

[B38] Kaul S. Sharma T. Dhar M. K. (2016). “Omics” Tools for better understanding the plant–endophyte interactions. *Front. Plant Sci.* 7:955. 10.3389/fpls.2016.00955 27446181 PMC4925718

[B39] Kõiv V. Roosaare M. Vedler E. Ann Kivistik P. Toppi K. Schryer D. W. (2015). Microbial population dynamics in response to *Pectobacterium atrosepticum* infection in potato tubers. *Sci. Rep.* 5:11606. 10.1038/srep11606 26118792 PMC4484245

[B40] Kuczynski J. Stombaugh J. Walters W. A. González A. Caporaso J. G. Knight R. (2011). Using QIIME to analyze 16S rRNA gene sequences from microbial communities. *Curr. Protoc. Bioinformatics* 36 10.7.1–10.7.20. 10.1002/0471250953.bi1007s36 22161565 PMC3249058

[B41] Langille M. G. I. Zaneveld J. Caporaso J. G. McDonald D. Knights D. Reyes J. A. (2013). Predictive functional profiling of microbial communities using 16S rRNA marker gene sequences. *Nat. Biotechnol.* 31 814–821. 10.1038/nbt.2676 23975157 PMC3819121

[B42] Larkin M. A. Blackshields G. Brown N. P. Chenna R. McGettigan P. A. McWilliam H. (2007). Clustal W and Clustal X version 2.0. *Bioinformatics* 23 2947–2948. 10.1093/bioinformatics/btm404 17846036

[B43] Lefort V. Longueville J.-E. Gascuel O. (2017). SMS: Smart Model Selection in PhyML. *Mol. Biol. Evol.* 34 2422–2424. 10.1093/molbev/msx149 28472384 PMC5850602

[B44] Letunic I. Bork P. (2007). Interactive tree of life (iTOL): an online tool for phylogenetic tree display and annotation. *Bioinformatics* 23 127–128. 10.1093/bioinformatics/btl529 17050570

[B45] Liu Y. H. Guo J. W. Salam N. Li L. Zhang Y. G. Han J. (2016). Culturable endophytic bacteria associated with medicinal plant *Ferula songorica*: molecular phylogeny, distribution and screening for industrially important traits. *3 Biotech* 6:209. 10.1007/s13205-016-0522-7 28330280 PMC5040645

[B46] Lòpez-Fernàndez S. Mazzoni V. Pedrazzoli F. Pertot I. Campisano A. (2017). A phloem-feeding insect transfers bacterial endophytic communities between grapevine plants. *Front. Microbiol.* 15:834. 10.3389/fmicb.2017.00834 28555131 PMC5430944

[B47] Lozupone C. Lladser M. E. Knights D. Stombaugh J. Knight R. (2011). UniFrac: an effective distance metric for microbial community comparison. *ISME J.* 5 169–172. 10.1038/ismej.2010.133 20827291 PMC3105689

[B48] Magurran E. (1998). *Ecological Diversity and its Measurement.* Princeton, NJ: Princeton University Press.

[B49] Mason C. J. Hanshew A. S. Raffa K. F. (2015). Contributions by host trees and insect activity to bacterial communities in *Dendroctonus valens* (Coleoptera: Curculionidae) galleries, and their high overlap with other microbial assemblages of bark beetles. *Environ. Entomol.* 45 348–356. 10.1093/ee/nvv184 26721298

[B50] Mendes R. Pizzirani-Kleiner A. A. Araujo W. L. Raaijmakers J. M. (2007). Diversity of cultivated endophytic bacteria from sugarcane: genetic and biochemical characterization of *Burkholderia cepacia* complex isolates. *Appl. Environ. Microbiol.* 73 7259–7267. 10.1128/AEM.01222-07 17905875 PMC2168197

[B51] Mendoza M. G. Salinas-Moreno Y. Olivo-Martínez A. Zúñiga G. (2011). Factors influencing the geographical distribution of *Dendroctonus rhizophagus* (Coleoptera: Curculionidae: Scolytinae) in the sierra madre occidental, Mexico. *Environ. Entomol.* 40 549–559. 10.1603/EN10059 22251632

[B52] Morales-Jiménez J. Vera-Ponce de León A. García-Domínguez A. Martínez-Romero E. Zúñiga G. Hernández-Rodríguez C. (2013). Nitrogen-fixing and uricolytic bacteria associated with the gut of *Dendroctonus rhizophagus* and *Dendroctonus valens* (Curculionidae: Scolytinae). *Microb. Ecol.* 66 200–210. 10.1007/s00248-013-0206-3 23525792

[B53] Morales-Jiménez J. Zúñiga G. Ramírez-Saad H. C. Hernández-Rodríguez C. (2012). Gut-associated bacteria throughout the life cycle of the bark beetle *Dendroctonus rhizophagus* Thomas and Bright (Curculionidae: Scolytinae) and their cellulolytic activities. *Microb. Ecol.* 64 268–278. 10.1007/s00248-011-9999-0 22234511

[B54] Morales-Jiménez J. Zúñiga G. Villa-Tanaca L. Hernández-Rodríguez C. (2009). Bacterial community and nitrogen fixation in the red turpentine beetle, *Dendroctonus valens* LeConte (Coleoptera: Curculionidae: Scolytinae). *Microb. Ecol.* 58 879–891. 10.1007/s00248-009-9548-2 19543937

[B55] Morris C. E. (2002). *Phyllosphere.* Hoboken, NJ: John Wiley & Sons.

[B56] Moyes A. B. Kueppers L. M. Pett-Ridge J. Carper D. L. Vandehey N. O’Neil J. (2016). Evidence for foliar endophytic nitrogen fixation in a widely distributed subalpine conifer. *New Phytol.* 210 657–668. 10.1111/nph.13850 27000956

[B57] Navarro-Noya Y. E. Suárez-Arriaga M. C. Rojas-Valdes A. Montoya-Ciriaco N. M. Gómez-Acata S. Fernández-Luqueño F. (2013). Pyrosequencing analysis of the bacterial community in drinking water wells. *Microb. Ecol.* 66 19–29. 10.1007/s00248-013-0222-3 23563631

[B58] Oliveros J. C. (2007). *VENNY. An Interactive Tool for Comparing Lists with Venn Diagrams.* Available at: http://bioinfogp.cnb.csic.es/tools/venny/index.html

[B59] O’Neill G. A. Chanway C. P. Axelrood P. E. Radley R. A. Holl F. B. (1992). An assessment of spruce growth response specificity after inoculation with coexistent rhizosphere bacteria. *Can. J. Bot.* 70 2347–2353. 10.1139/b92-294

[B60] Parmentier F. J. W. Van Huissteden J. Kip N. Op Den Camp H. J. M. Jetten M. S. M. Maximov T. C. (2011). The role of endophytic methane-oxidizing bacteria in submerged *Sphagnum* in determining methane emissions of Northeastern Siberian tundra. *Biogeosciences* 8 1267–1278. 10.5194/bg-8-1267-2011

[B61] Pirttilä A. M. Laukkanen H. Pospiech H. Myllylä R. Hohtola A. (2000). Detection of intracellular bacteria in the buds of scotch pine (*Pinus sylvestris* L.) by in situ hybridization. *Appl. Environ. Microbiol.* 66 3073–3077. 10.1128/AEM.66.7.3073-3077.2000 10877808 PMC92113

[B62] Price M. N. Dehal P. S. Arkin A. P. (2009). FastTree: computing large minimum evolution trees with profiles instead of a distance matrix. *Mol. Biol. Evol.* 26 1641–1650. 10.1093/molbev/msp077 19377059 PMC2693737

[B63] Raffa K. F. Aukema B. H. Bentz B. J. Carroll A. L. Hicke J. A. Turner M. G. (2008). Cross-scale drivers of natural disturbances prone to anthropogenic amplification: the dynamics of bark beetle eruptions. *Bioscience* 58 501–517. 10.1641/B580607

[B64] Reeve J. D. Anderson F. E. Kelley S. T. (2012). Ancestral state reconstruction for *Dendroctonus* bark beetles: evolution of a tree killer. *Environ. Entomol.* 41 723–730. 10.1603/EN11281 22732632

[B65] Reinhold-Hurek B. Hurek T. (2011). Living inside plants: bacterial endophytes. *Curr. Opin. Plant Biol.* 14 435–443. 10.1016/j.pbi.2011.04.004 21536480

[B66] Reiter B. Pfeifer U. Schwab H. Sessitsch A. (2002). Response of endophytic bacterial communities in potato plants to infection with *Erwinia carotovora* subsp. atroseptica. *Appl. Environ. Microbiol.* 68 2261–2268. 10.1128/aem.68.5.2261-2268.2002 11976096 PMC127529

[B67] Rohlf F. J. (1997). *NTSYSpc Numerical Taxonomy and Multivariate Analysis System Version 2.0 User Guide.* East Setauket, NY: Applied Biostatistics Inc.

[B68] Rúa M. A. Wilson E. C. Steele S. Munters A. R. Hoeksema J. D. Frank A. C. (2016). Associations between ectomycorrhizal fungi and bacterial needle endophytes in *Pinus radiata*: implications for biotic selection of microbial communities. *Front. Microbiol.* 7:399. 10.3389/fmicb.2016.00399 27065966 PMC4815291

[B69] Ruiz-Pérez C. A. Restrepo S. Zambrano M. M. (2016). Microbial and functional diversity within the phyllosphere of *Espeletia* species in an Andean high-mountain ecosystem. *Appl. Environ. Microbiol.* 82 1807–1817. 10.1128/AEM.02781-15 26746719 PMC4784022

[B70] Salinas-Moreno Y. Mendoza M. G. Barrios M. A. Cisneros R. Macias-Samano J. Zuniga G. (2004). Aerography of the genus *Dendroctonus* (Coleoptera: Curculionidae: Scolytinae) in Mexico. *J. Biogeogr.* 31 1163–1177. 10.1111/j.1365-2699.2004.01110.x

[B71] Sánchez-López A. S. Thijs S. Beckers B. González-Chávez M. C. Weyens N. Carrillo-González R. (2017). Community structure and diversity of endophytic bacteria in seeds of three consecutive generations of *Crotalaria pumila* growing on metal mine residues. *Plant Soil* 25 1–16. 10.1007/s11104-017-3176-2

[B72] Scott J. J. Oh D. C. Yuceer M. C. Klepzig K. D. Clardy J. Currie C. R. (2008). Bacterial protection of beetle-fungus mutualism. *Science* 322:63. 10.1126/science.1160423 18832638 PMC2761720

[B73] Scully E. D. Geib S. M. Hoover K. Tien M. Tringe S. G. Barry K. W. (2013). Metagenomic profiling reveals lignocellulose degrading system in a microbial community associated with a wood-feeding beetle. *PLOS ONE* 8:e73827. 10.1371/journal.pone.0073827 24023907 PMC3762729

[B74] Sessitsch A. Reiter B. Pfeifer U. Wilhelm E. (2002). Cultivation-independent population analysis of bacterial endophytes in three potato varieties based on eubacterial and actinomycetes-specific PCR of 16S rRNA genes. *FEMS Microbiol. Ecol.* 39 23–32. 10.1111/j.1574-6941.2002.tb00903.x 19709181

[B75] Sheibani-Tezerji R. Rattei T. Sessitsch A. Trognitz F. Mitter B. (2015). Transcriptome profiling of the endophyte *Burkholderia phytofirmans* PsJN indicates sensing of the plant environment and drought stress. *mBio* 6 e00621–15. 10.1128/mBio.00621-15 26350963 PMC4600099

[B76] Shishido M. Loeb B. M. Chanway C. P. (1995). External and internal root colonization of lodgepole pine seedlings by two growth-promoting Bacillus strains originated from different root microsites. *Can. J. Microbiol.* 41 707–713. 10.1139/m95-097

[B77] Strzelczyk E. Li C. Y. (2000). Bacterial endobionts in the big non-mycorrhizal roots of Scots pine (*Pinus sylvestris* L.). *Microbiol. Res.* 155 229–232. 10.1016/S0944-5013(00)80037-3 11061192

[B78] Sturz A. V. Christie B. R. Matheson B. G. (1998). Associations of bacterial endophyte populations from red clover and potato crops with potential for beneficial allelopathy. *Can. J. Microbiol.* 44 162–167. 10.1139/w97-146

[B79] Sun L. Qiu F. Zhang X. Dai X. Dong X. Song W. (2008). Endophytic bacterial diversity in rice (*Oryza sativa* L.) roots estimated by 16S rDNA sequence analysis. *Microb. Ecol.* 55 415–424. 10.1007/s00248-007-9287-1 17690836

[B80] Turner T. R. James E. K. Poole P. S. Gilbert J. Meyer F. Jansson J. (2013). The plant microbiome. *Genome Biol.* 14:209. 10.1186/gb-2013-14-6-209 23805896 PMC3706808

[B81] Van Aken B. Peres C. M. Doty S. L. Yoon J. M. Schnoor J. L. (2004). *Methylobacterium populi* sp. nov., a novel aerobic, pink-pigmented, facultatively methylotrophic, methane-utilizing bacterium isolated from poplar trees (*Populus deltoides x nigra* DN34). *Int. J. Syst. Evol. Microbiol.* 54 1191–1196. 10.1099/ijs.0.02796-0 15280290

[B82] Vandenkoornhuyse P. Quaiser A. Duhamel M. Le Van A. Dufresne A. (2015). The importance of the microbiome of the plant holobiont. *New Phytol.* 206 1196–1206. 10.1111/nph.13312 25655016

[B83] Wang Q. Garrity G. M. Tiedje J. M. Cole J. R. (2007). Naïve Bayesian classifier for rapid assignment of rRNA sequences into the new bacterial taxonomy. *Appl. Environ. Microbiol.* 73 5261–5267. 10.1128/AEM.00062-07 17586664 PMC1950982

[B84] Whipps J. M. Hand P. Pink D. Bending G. D. (2008). Phyllosphere microbiology with special reference to diversity and plant genotype. *J. Appl. Microbiol.* 105 1744–1755. 10.1111/j.1365-2672.2008.03906.x 19120625

[B85] Wood D. (1982). The role of pheromones, kairomones, and allomones in the host selection and colonization behavior of bark beetles. *Annu. Rev. Entomol.* 27 411–446. 10.1146/annurev.en.27.010182.002211

[B86] Xia Y. DeBolt S. Dreyer J. Scott D. Williams M. A. (2015). Characterization of culturable bacterial endophytes and their capacity to promote plant growth from plants grown using organic or conventional practices. *Front. Plant Sci.* 6:490. 10.3389/fpls.2015.00490 26217348 PMC4498380

[B87] Xu L. T. Lu M. Sun J. H. (2016). Invasive bark beetle-associated microbes degrade a host defensive monoterpene. *Insect Sci.* 23 183–190. 10.1111/1744-7917.12255 26224144

[B88] Xu L. Shi Z. Wang B. Lu M. Sun J. H. (2016). Pine defensive monoterpene α-pinene influences the feeding behavior of *Dendroctonus valens* and its gut bacterial community structure. *Int. J. Mol. Sci.* 17:1734. 10.3390/ijms17111734 27809267 PMC5133772

[B89] Yang R. Liu P. Ye W. (2017). Illumina-based analysis of endophytic bacterial diversity of tree peony (*Paeonia* Sect. Moutan) roots and leaves. *Braz. J. Microbiol.* 48 695–705. 10.1016/j.bjm.2017.02.009 28606427 PMC5628320

[B90] Zarraonaindia I. Owens S. M. Weisenhorn P. West K. Hampton-marcell J. Lax S. (2015). The soil microbiome influences grapevine-associated microbiota. *MBio* 6 e02527-14. 10.1128/mBio.02527-14 25805735 PMC4453523

[B91] Zhou J. Bruns M. A. Tiedje J. M. (1996). DNA recovery from soils of diverse composition. *Appl. Environ. Microbiol.* 62:316–322. 8593035 10.1128/aem.62.2.316-322.1996 PMC167800

[B92] Zinniel D. K. Lambrecht P. Harris N. B. Kuczmarski D. Higley P. Ishimaru C. A. (2002). Isolation and characterization of endophytic colonizing bacteria from agronomic crops and prairie plants isolation and characterization of endophytic colonizing bacteria from agronomic crops and prairie plants. *Appl. Environ. Microbiol.* 68 2198–2208. 10.1128/AEM.68.5.2198 11976089 PMC127535

