# Supplementary material for: Bacteriome from Pinus arizonica and P. durangensis: Diversity, Comparison of Assemblages, and Overlapping Degree with the Gut Bacterial Community of a Bark Beetle That Kills Pines
Source: Front Microbiol. 2018 Jan 30;9:77. doi: 10.3389/fmicb.2018.00077 (PMC5797664; doi:10.3389/fmicb.2018.00077)
Supplement: Supplementary file 1 [file Data_Sheet_1.docx]

Supplementary Material

**Bacteriome from *Pinus arizonica* and *P. durangensis*: diversity, comparison of assemblages, and overlapping degree with the gut bacterial community of a bark beetle that kills pines**

**Roman Gonzalez-Escobedo, Carlos Iván Briones-Roblero, Rosa María Pineda-Mendoza, Flor N. Rivera-Orduña, and Gerardo Zúñiga**

# Supplementary Figures

**Supplementary Figure S1.** Distribution of relative abundance of endophytic bacterial associated with root, phloem, and bark of Durango and Arizona pines at different taxonomic levels. **(A)** phyla, and **(B)** family (RootPD1 = Root *P. durangensis*1, RootPD2 = Root *P. durangensis*2, PhloemPD1 = Phloem *P. durangensis*1, PhloemPD2 = Phloem *P. durangensis*2, BarkPD1 = Bark *P. durangensis*1, BarkPD2 = Bark *P. durangensis*2; RootPA1 = Root *P. arizonica*1, RootPA2 = Root *P. arizonica*2, PhloemPA1 = Phloem *P. arizonica*1, PhloemPA2 = Phloem *P. arizonica*2, BarkPA1 = Bark *P. arizonica*1).

**Supplementary Figure S2.** Rarefaction curves of samples of Durango and Arizona pines (RootPD1 = Root *P. durangensis*1, RootPD2 = Root *P. durangensis*2, PhloemPD1 = Phloem *P. durangensis*1, PhloemPD2 = Phloem *P. durangensis*2, BarkPD1 = Bark *P. durangensis*1, BarkPD2 = Bark *P. durangensis*2; RootPA1 = Root *P. arizonica*1, RootPA2 = Root *P. arizonica*2, PhloemPA1 = Phloem *P. arizonica*1, PhloemPA2 = Phloem *P. arizonica*2, BarkPA1 = Bark *P. arizonica*1).

**Supplementary Figure S3**. Maximum likelihood phylogenetic trees with sequences of pyrosequenced bacterial sequences of this study (red), gut bacterial sequences of *D. rhizophagus* (green), GenBank sequences of isolated bacteria of *D. rhizophagus* (blue), and reference sequences of GenBank. **(A)** *Pseudomonas*, **(B)** *Rahnella*, **(C)** *Serratia*, **(D)** *Propionibacterium*, **(E)** *Stenotrophomonas*, and **(F)** *Acinetobacter*.

**Supplementary Figure S4**. Abundances of KEGG pathways in level-3 of the functional prediction by PICRUSt of Durango and Arizona pines. **(A)** Lipid metabolism, glycan biosynthesis and metabolism, energy metabolism, carbohydrate metabolism, biosynthesis of other secondary metabolites and amino acid metabolism, and **(B)** Xenobiotic biodegradation and metabolism, metabolism of terpenoids and polyketides, metabolism of other amino acids and metabolism of cofactor and vitamins (RootPD1 = Root *P. durangensis*1, RootPD2 = Root *P. durangensis*2, PhloemPD1 = Phloem *P. durangensis*1, PhloemPD2 = Phloem *P. durangensis*2, BarkPD1 = Bark *P. durangensis*1, BarkPD2 = Bark *P. durangensis*2; RootPA1 = Root *P. arizonica*1, RootPA2 = Root *P. arizonica*2, PhloemPA1 = Phloem *P. arizonica*1, PhloemPA2 = Phloem *P. arizonica*2, BarkPA1 = Bark *P. arizonica*1).

**Supplementary Figure S5.** Heatmap depicting the PICRUSt-inferred gene relative abundance in the predicted gut bacterial communities of different *Dendroctonus* species. Warm colors represent high abundances and clear colors represent low abundances (DADJ, *D. adjunctus*; DAPP, *D. approximatus*; DBRE, *D. brevicomis*; DFRO, *D. frontalis*; DJEF, *D. jeffreyi*; DMES, *D. mesoamericanus*; DMEX, *D. mexicanus*; DPAR, *D. parallelocollis*; DPON, *D. ponderosae*; DPSE, *D. pseudotsugae*; DRHI, *D. rhizophagus*; DVAL, *D. valens*; DVIT, *D. vitei*).


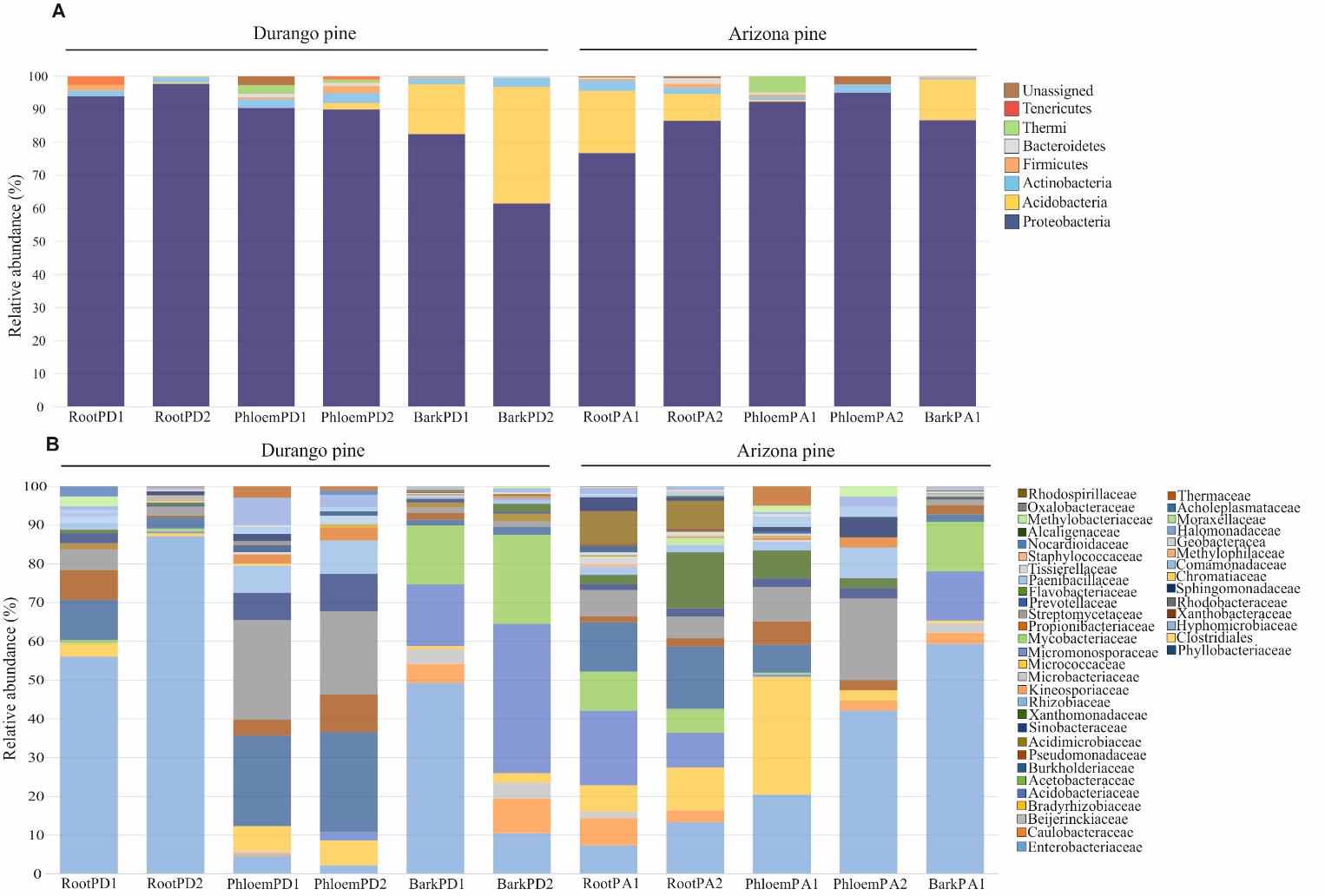


**Supplementary Figure S1.**


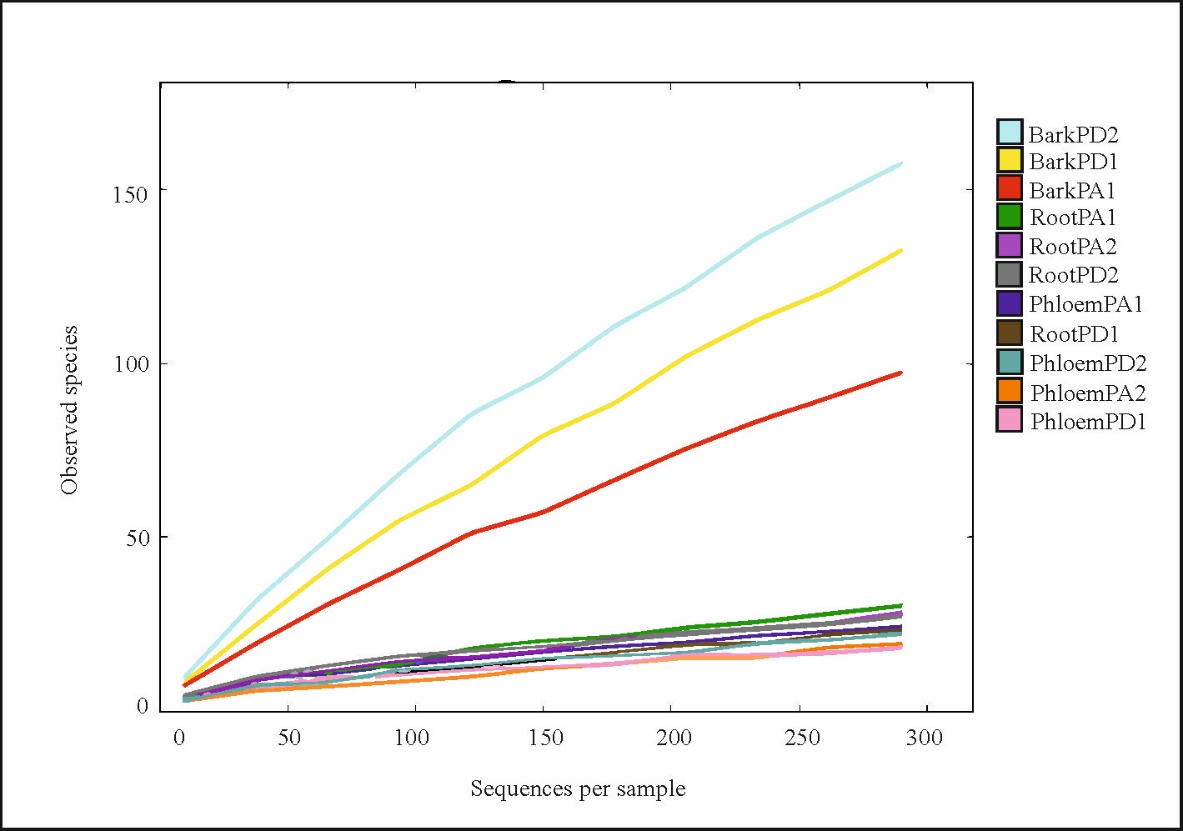


**Supplementary Figure S2.**


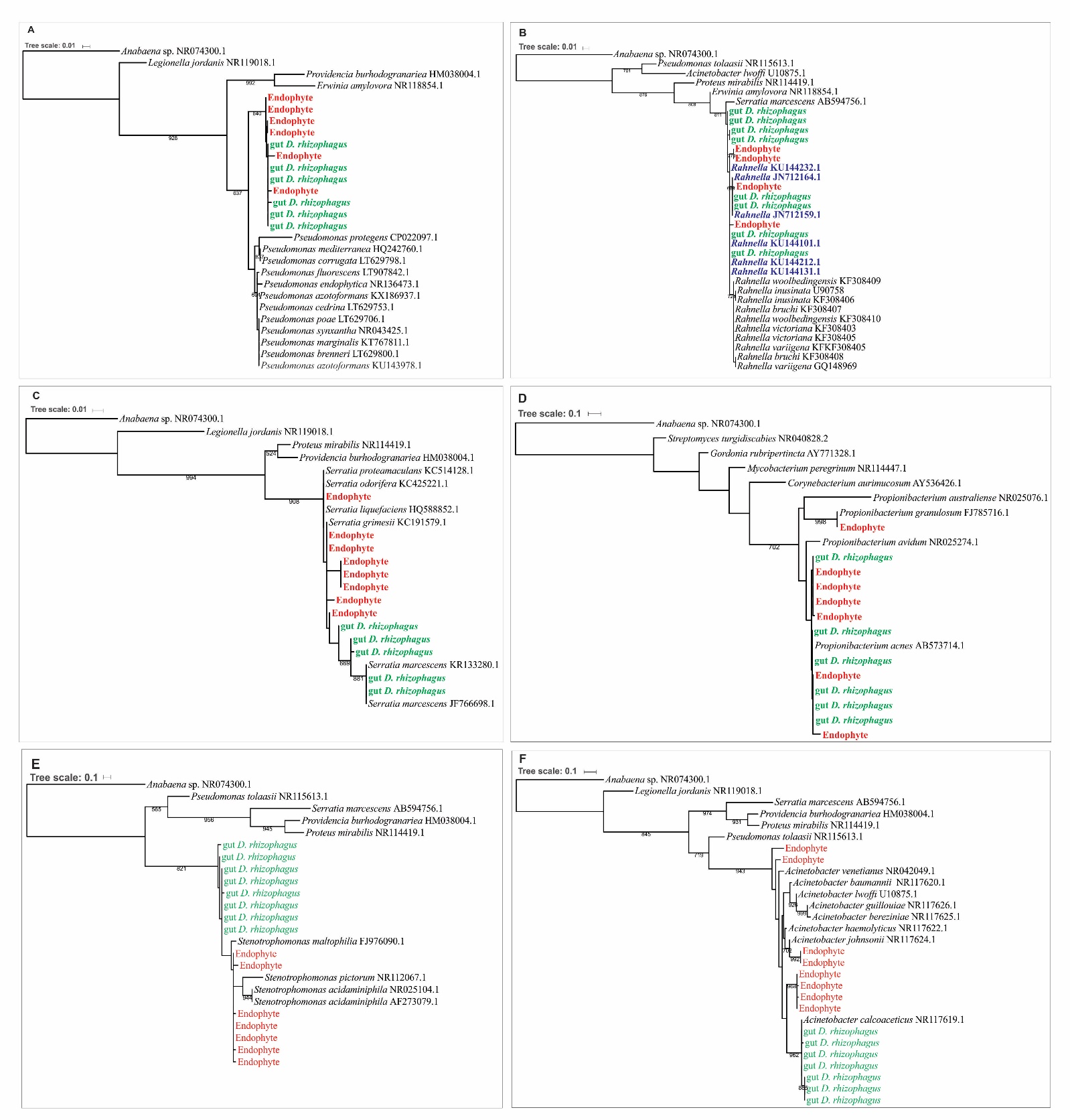


**Supplementary Figure S3.**


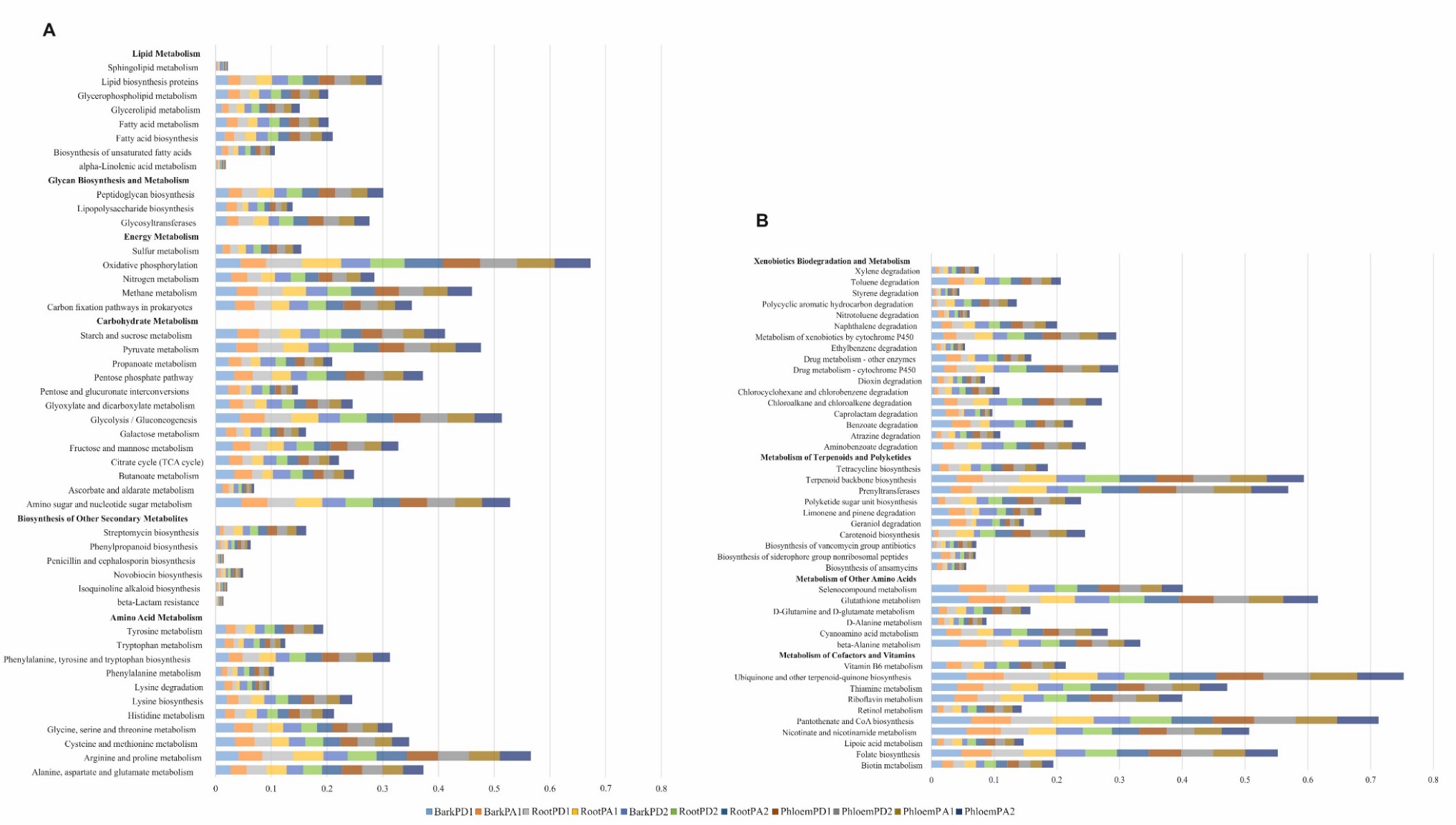


**Supplementary Figure S4.**

#
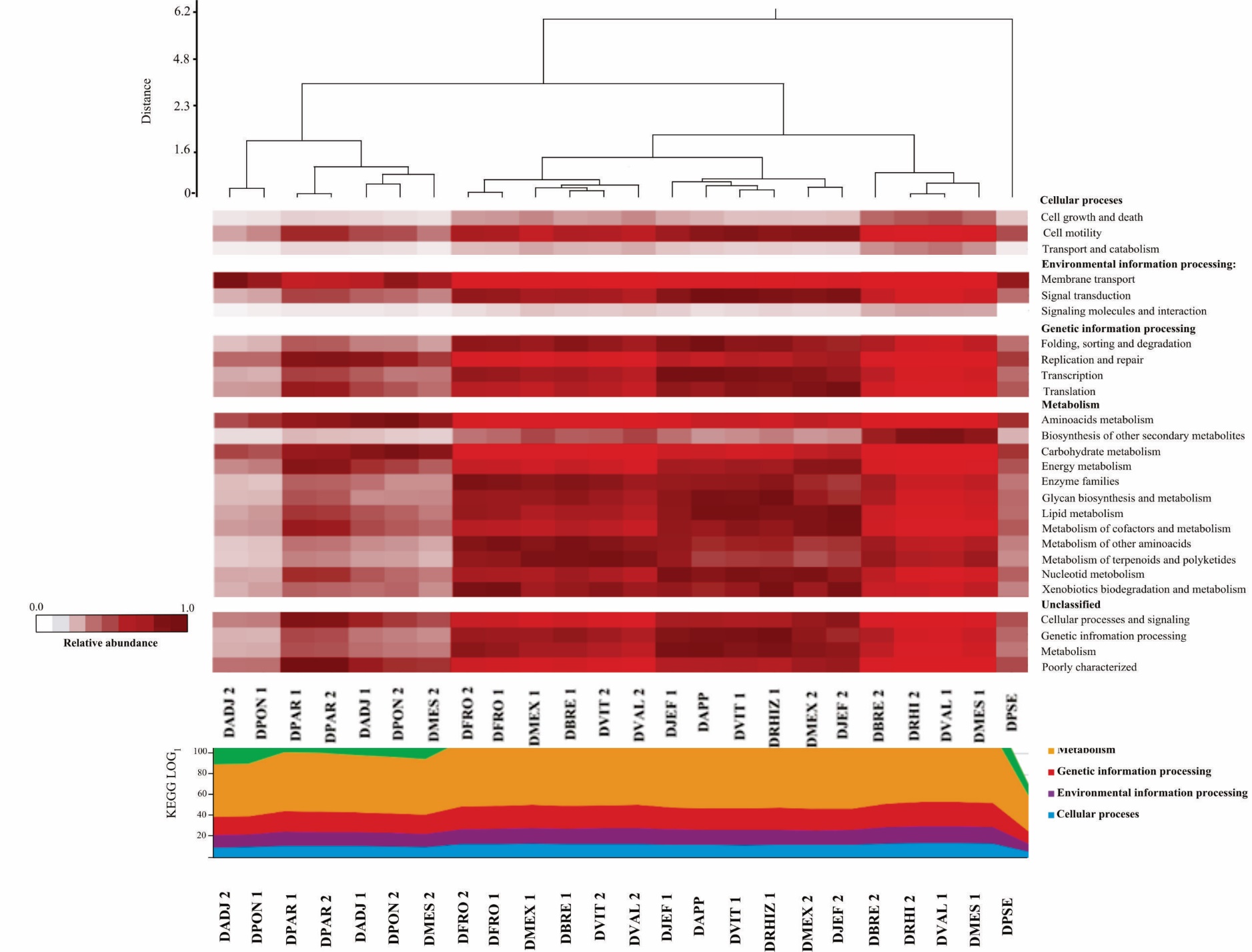


**Supplementary Figure S5.**

# Supplementary Tables

**Supplementary Table S1.** Taxonomic assignment of endophytic bacterial communities from Durango and Arizona pines.

| **Phylum** | **Class** | **Order** | **Family** | **Genus** |
| --- | --- | --- | --- | --- |
| Acidobacteria | Acidobacteria | Acidobacteriales | Acidobacteriaceae | Unclassified |
| Thermi | Deinococci | Thermales | Thermacea | *Meiothermus* |
| Actinobacteria | Actinobacteria | Acidimicrobiales | Acidimicrobiaceae | Unclassified |
| - | - | Actinomycetales | Kineosporiaceae | Unclassified |
| - | - | - | Microbacteriaceae | *Leucobacter* |
| - | - | - | Micrococcaceae | *Arthrobacter* |
| - | - | - | - | *Kocuria* |
| - | - | - | Micromonosporaceae | Unclassified |
| - | - | - | Mycobacteriaceae | *Mycobacterium* |
| - | - | - | Nocardioidaceae | *Friedmanniella* |
| - | - | - | Propionibacteriaceae | *Propionibacterium* |
| - | - | - | Streptomycetaceae | *Streptomyces* |
| - | Thermoleophilia | Solirubrobacterales | Unclassified | Unclassified |
| Bacteroidetes | Bacteroidia | Bacteroidales | Prevotellaceae | *Prevotella* |
| - | Flavobacteriia | Flavobacteriales | Flavobacteriaceae | *Capnocytophaga* |
| - | Sphingobacteriia | Sphingobacteriales | Unclassified | Unclassified |
| Firmicutes | Bacilli | Bacillales | Paenibacillaceae | *Ammoniphilus* |
| - | - | - | Staphylococcaceae | *Staphylococcus* |
| - | Clostridia | Clostridiales | Tissierellaceae | *Peptoniphilus* |
| - | - | - | Clostridiales Family XI. Incertae Sedis | Unclassified |
| Proteobacteria | Alphaproteobacteria | Caulobacterales | Caulobacteraceae | *Brevundimonas* |
| - | - | - | - | *Caulobacter* |
| - | - | Rhizobiales | Beijerinckiaceae | *Methylocapsa* |
| - | - | - | Bradyrhizobiaceae | *Bradyrhizobium* |
| - | - | - | Hyphomicrobiaceae | *Hyphomicrobium* |
| - | - | - | Methylobacteriaceae | *Methylobacterium* |
| - | - | - | Phyllobacteriaceae | *Mesorhizobium* |
| - | - | - | Rhizobiaceae | *Rhizobium* |
| - | - | - | Xanthobacteraceae | *Labrys* |
| - | - | Rhodobacterales | Rhodobacteraceae | *Paracoccus* |
| - | - | Rhodospirillales | Acetobacteraceae | *Acetobacter* |
| - | - | - | Rhodospirillaceae | Unclassified |
| - | - | Sphingomonadales | Sphingomonadaceae | *Novosphingobium* |
| - | - | - | - | *Sphingomonas* |
| - | Betaproteobacteria | Burkholderiales | Alcaligenaceae | *Achromobacter* |
| - | - | - | Burkholderiaceae | *Burkholderia* |
| - | - | - | Comamonadaceae | *Comamonas* |
| - | - | - | - | *Delftia* |
| - | - | - | - | *Pelomonas* |
| - | - | - | Oxalobacteraceae | *Herbaspirillum* |
| - | - | - | - | *Ralstonia* |
| - | - | Methylophilales | Methylophilaceae | *Methylophilus* |
| - | Deltaproteobacteria | Desulfovibrionales | Unclassified | Unclassified |
| - | - | Desulfuromonadales | Geobacteracea | Unclassified |
| - | - | Myxococcales | Unclassified | Unclassified |
| - | Gammaproteobacteria | Alteromonadales | Chromatiaceae | *Rheinheimera* |
| - | - | Enterobacteriales | Enterobacteriaceae | *Citrobacter* |
| - | - | - | - | *Klebsiella* |
| - | - | - | - | *Enterobacter* |
| - | - | - | - | *Pantoeae* |
| - | - | - | - | *Providencia* |
| - | - | - | - | *Rahnella* |
| - | - | - | - | *Salmonella* |
| - | - | - | - | *Serratia* |
| - | - | - | - | *Shigella* |
| - | - | Oceanospirillales | Halomonadaceae | *Halomonas* |
| - | - | Pseudomonadales | Pseudomonadaceae | *Pseudomonas* |
| - | - | - | Moraxellaceae | *Acinetobacter* |
| - | - | Xanthomonadales | Sinobacteraceae | Unclassified |
| - | - | - | Xanthomonadaceae | *Dyella* |
| - | - | - | - | *Luteibacter* |
| - | - | - | - | *Stenotrophomonas* |
| Tenericutes | Mollicutes | Acholeplasmatales | Acholeplasmataceae | Ca. *Phytoplasma* |

**Supplementary Table S2.** Venn diagram details.

| ***Pinus arizonica* vs. *Pinus durangensis*** | | | | |  |  |
| --- | --- | --- | --- | --- | --- | --- |
| **Names** | **Total** | | **Elements** | |  |  |
| *Pinus arizonica* and *P. durangensis* | 39 | | *Mesorhizobium* | |  |  |
|  |  | | *Rheinheimera* | |  |  |
|  |  | | *Achromobacter* | |  |  |
|  |  | | *Methylobacterium* | |  |  |
|  |  | | *Serratia* | |  |  |
|  |  | | *Hyphomicrobium* | |  |  |
|  |  | | *Kocuria* | |  |  |
|  |  | | *Citrobacter* | |  |  |
|  |  | | *Methylophilus* | |  |  |
|  |  | | *Ralstonia* | |  |  |
|  |  | | *Brevundimonas* | |  |  |
|  |  | | *Staphylococcus* | |  |  |
|  |  | | *Pelomonas* | |  |  |
|  |  | | *Meiothermus* | |  |  |
|  |  | | *Burkholderia* | |  |  |
|  |  | | *Acetobacter* | |  |  |
|  |  | | *Propionibacterium* | |  |  |
|  |  | | *Paracoccus* | |  |  |
|  |  | | *Streptomyces* | |  |  |
|  |  | | *Ammoniphilus* | |  |  |
|  |  | | *Herbaspirillum* | |  |  |
|  |  | | *Pseudomonas* | |  |  |
|  |  | | *Providencia* | |  |  |
|  |  | | *Comamonas* | |  |  |
|  |  | | *Prevotella* | |  |  |
|  |  | | *Caulobacter* | |  |  |
|  |  | | *Bradyrhizobium* | |  |  |
|  |  | | *Salmonella* | |  |  |
|  |  | | *Shigella* | |  |  |
|  |  | | *Delftia* | |  |  |
|  |  | | *Sphingomonas* | |  |  |
|  |  | | *Acinetobacter* | |  |  |
|  |  | | *Rhizobium* | |  |  |
|  |  | | *Halomonas* | |  |  |
|  |  | | *Stenotrophomonas* | |  |  |
|  |  | | *Peptoniphilus* | |  |  |
|  |  | | *Rahnella* | |  |  |
|  |  | | *Leucobacter* | |  |  |
|  |  | | *Methylocapsa* | |  |  |
| *Pinus durangensis* | 6 | | *Klebsiella* | |  |  |
|  |  | | Ca. *Phytoplasma* | |  |  |
|  |  | | *Capnocytophaga* | |  |  |
|  |  | | *Pantoeae* | |  |  |
|  |  | | *Enterobacter* | |  |  |
|  |  | | *Friedmaniella* | |  |  |
| *Pinus arizonica* | 6 | | *Arthrobacter* | |  |  |
|  |  | | *Novosphingobium* | |  |  |
|  |  | | *Dyella* | |  |  |
|  |  | | *Luteibacter* | |  |  |
|  |  | | *Labrys* | |  |  |
|  |  | | *Mycobacterium* | |  |  |
| ***Pinus arizonica*** | | | | |  |  |
| **Names** | **Total** | | **Elements** | |  |  |
| Bark, phloem, root | 12 | | *Mesorhizobium* | |  |  |
|  |  | | *Serratia* | |  |  |
|  |  | | *Ralstonia* | |  |  |
|  |  | | *Burkholderia* | |  |  |
|  |  | | *Propionibacterium* | |  |  |
|  |  | | *Pseudomonas* | |  |  |
|  |  | | *Bradyrhizobium* | |  |  |
|  |  | | *Delftia* | |  |  |
|  |  | | *Acinetobacter* | |  |  |
|  |  | | *Rhizobium* | |  |  |
|  |  | | *Halomonas* | |  |  |
|  |  | | *Stenotrophomonas* | |  |  |
| Phloem, root | 1 | | *Rahnella* | |  |  |
| Bark, root | 9 | | *Hyphomicrobium* | |  |  |
|  |  | | *Pelomonas* | |  |  |
|  |  | | *Ammoniphilus* | |  |  |
|  |  | | *Herbaspirillum* | |  |  |
|  |  | | *Providencia* | |  |  |
|  |  | | *Dyella* | |  |  |
|  |  | | *Caulobacter* | |  |  |
|  |  | | *Peptoniphilus* | |  |  |
|  |  | | *Methylocapsa* | |  |  |
| Bark, phloem | 3 | | *Citrobacter* | |  |  |
|  |  | | *Brevundimonas* | |  |  |
|  |  | | *Meiothermus* | |  |  |
| Root | 7 | | *Achromobacter* | |  |  |
|  |  | | *Methylophilus* | |  |  |
|  |  | | *Novosphingobium* | |  |  |
|  |  | | *Streptomyces* | |  |  |
|  |  | | *Luteibacter* | |  |  |
|  |  | | *Labrys* | |  |  |
|  |  | | *Mycobacterium* | |  |  |
| Phloem | 1 | | *Leucobacter* | |  |  |
| Bark | 11 | | *Methylobacterium* | |  |  |
|  |  | | *Kocuria* | |  |  |
|  |  | | *Arthrobacter* | |  |  |
|  |  | | *Staphylococcus* | |  |  |
|  |  | | *Acetobacter* | |  |  |
|  |  | | *Paracoccus* | |  |  |
|  |  | | *Comamonas* | |  |  |
|  |  | | *Prevotella* | |  |  |
|  |  | | *Salmonella* | |  |  |
|  |  | | *Shigella* | |  |  |
|  |  | | *Sphingomonas* | |  |  |
| ***Pinus durangensis*** | | | | | |  |
| **Names** | **Total** | | **Elements** | | |  |
| Bark, phloem, root | 15 | | *Klebsiella* | | |  |
|  |  | | Ca. *Phytoplasma* | | |  |
|  |  | | *Serratia* | | |  |
|  |  | | *Kocuria* | | |  |
|  |  | | *Ralstonia* | | |  |
|  |  | | *Pelomonas* | | |  |
|  |  | | *Meiothermus* | | |  |
|  |  | | *Burkholderia* | | |  |
|  |  | | *Propionibacterium* | | |  |
|  |  | | *Pseudomonas* | | |  |
|  |  | | *Providencia* | | |  |
|  |  | | *Bradyrhizobium* | | |  |
|  |  | | *Rhizobium* | | |  |
|  |  | | *Halomonas* | | |  |
|  |  | | *Rahnella* | | |  |
| Bark, root | 14 | | *Hyphomicrobium* | | |  |
|  |  | | *Citrobacter* | | |  |
|  |  | | *Methylophilus* | | |  |
|  |  | | *Brevundimonas* | | |  |
|  |  | | *Staphylococcus* | | |  |
|  |  | | *Acetobacter* | | |  |
|  |  | | *Ammoniphilus* | | |  |
|  |  | | *Herbaspirillum* | | |  |
|  |  | | *Delftia* | | |  |
|  |  | | *Sphingomonas* | | |  |
|  |  | | *Acinetobacter* | | |  |
|  |  | | *Stenotrophomonas* | | |  |
|  |  | | *Enterobacter* | | |  |
|  |  | | *Pantoeae* | | |  |
| Bark, phloem | 4 | | *Mesorhizobium* | | |  |
|  |  | | *Paracoccus* | | |  |
|  |  | | *Caulobacter* | | |  |
|  |  | | *Peptoniphilus* | | |  |
| Root | 1 | | *Streptomyces* | | |  |
| Bark | 11 | | *Rheinheimera* | | |  |
|  |  | | *Achromobacter* | | |  |
|  |  | | *Methylobacterium* | | |  |
|  |  | | *Comamonas* | | |  |
|  |  | | *Prevotella* | | |  |
|  |  | | *Salmonella* | | |  |
|  |  | | *Shigella* | | |  |
|  |  | | *Capnocytophaga* | | |  |
|  |  | | *Leucobacter* | | |  |
|  |  | | *Methylocapsa* | | |  |
|  |  | | *Friedmaniella* | | |  |
| ***Pinus arizonica* and *P. durangensis* vs *D. rhizophagus*** | | | | | | |
| **Names** | | **Total** | | **Elements** | | |
| *Pinus arizonica, P. durangensis*, *D. rhizophagus* | | 13 | | *Kocuria* | | |
|  | |  | | *Prevotella* | | |
|  | |  | | *Propionibacterium* | | |
|  | |  | | *Methylobacterium* | | |
|  | |  | | *Pseudomonas* | | |
|  | |  | | *Stenotrophomonas* | | |
|  | |  | | *Enterobacter* | | |
|  | |  | | *Acinetobacter* | | |
|  | |  | | *Burkholderia* | | |
|  | |  | | *Providencia* | | |
|  | |  | | *Pantoea* | | |
|  | |  | | *Serratia* | | |
|  | |  | | *Rahnella* | | |
| *D. rhizophagus* | | 10 | | *Anaerococcus* | | |
|  | |  | | *Streptococcus* | | |
|  | |  | | *Carnobacterium* | | |
|  | |  | | *Pseudoxanthomonas* | | |
|  | |  | | *Shewanella* | | |
|  | |  | | *Raoultella* | | |
|  | |  | | *Lactococcus* | | |
|  | |  | | *Corynebacterium* | | |
|  | |  | | *Bacillus* | | |
|  | |  | | *Proteus* | | |
| *Pinus arizonica* and *P. durangensis* | | 38 | | *Meiothermus* | | |
|  | |  | | *Acetobacter* | | |
|  | |  | | *Arthrobacter* | | |
|  | |  | | *Rhizobium* | | |
|  | |  | | *Methylophilus* | | |
|  | |  | | *Novosphingobium* | | |
|  | |  | | *Capnocytophaga* | | |
|  | |  | | *Leucobacter* | | |
|  | |  | | *Ammoniphilus* | | |
|  | |  | | *Brevundimonas* | | |
|  | |  | | *Sphingomonas* | | |
|  | |  | | *Salmonella* | | |
|  | |  | | *Paracoccus* | | |
|  | |  | | *Streptomyces* | | |
|  | |  | | *Comamonas* | | |
|  | |  | | *Labrys* | | |
|  | |  | | *Achromobacter* | | |
|  | |  | | *Dyella* | | |
|  | |  | | *Methylocapsa* | | |
|  | |  | | *Halomonas* | | |
|  | |  | | *Klebsiella* | | |
|  | |  | | *Mycobacterium* | | |
|  | |  | | *Ca. Phytoplasma* | | |
|  | |  | | *Bradyrhizobium* | | |
|  | |  | | *Ralstonia* | | |
|  | |  | | *Staphylococcus* | | |
|  | |  | | *Pelomonas* | | |
|  | |  | | *Caulobacter* | | |
|  | |  | | *Luteibacter* | | |
|  | |  | | *Herbaspirillum* | | |
|  | |  | | *Shigella* | | |
|  | |  | | *Friedmaniella* | | |
|  | |  | | *Hyphomicrobium* | | |
|  | |  | | *Peptoniphilus* | | |
|  | |  | | *Mesorhizobium* | | |
|  | |  | | *Delftia* | | |
|  | |  | | *Citrobacter* | | |
|  | |  | | *Rheinheimera* | | |

**Supplementary Table S3.** Correlation of PICRUSt functional abundances between endophytic bacterial and gut bacterial communities.

| KEGG_Pathways | PICRUSt data | | Spearman r | P-value |
| --- | --- | --- | --- | --- |
|  | Endophytes | Gut bacterial |  |  |
| Metabolism; Amino Acid Metabolism | 14979213 | 6668816 | 0.94 | 3.9273E-06 |
| Metabolism; Biosynthesis of Other Secondary Metabolites | 1779722 | 461092 |  |  |
| Metabolism; Carbohydrate Metabolism | 15684323 | 7829581 |  |  |
| Metabolism; Energy Metabolism | 17051178 | 3874695 |  |  |
| Metabolism; Enzyme Families | 4839901 | 1710398 |  |  |
| Metabolism; Glycan Biosynthesis and Metabolism | 3655987 | 1988573 |  |  |
| Metabolism; Lipid Metabolism | 4876877 | 2305904 |  |  |
| Metabolism; Metabolism of Cofactors and Vitamins | 10029201 | 3168740 |  |  |
| Metabolism; Metabolism of Other Amino Acids | 2910892 | 1319225 |  |  |
| Metabolism; Metabolism of Terpenoids and Polyketides | 3539126 | 1129601 |  |  |
| Metabolism; Nucleotide Metabolism | 5865706 | 2418454 |  |  |
| Metabolism; Xenobiotics Biodegradation and Metabolism | 3956704 | 1731940 |  |  |
